# Supplementary material for: Community treatment orders and associations with readmission rates and duration of psychiatric hospital admission: a controlled electronic case register study
Source: BMJ Open. 2020 Mar 5;10(3):e035121. doi: 10.1136/bmjopen-2019-035121 (PMC7059496; doi:10.1136/bmjopen-2019-035121)
Supplement: Supplementary data [file bmjopen-2019-035121supp001.pdf]

Community Treatment Orders: A controlled electronic case register study
Supplementary Material

Table S1 Number of patients in CTO and control group by year of study entry (*n* = 4,489)

**Year of study entry**	**Control group n (%)**	**CTO group n (%)**	**Total**
2008	560 (96.2%)	22 (3.8%)	582
2009	522 (81.3%)	120 (18.7%)	642
2010	503 (79.2%)	132 (20.8%)	635
2011	556 (80.6%)	134 (19.4%)	690
2012	615 (80.6%)	148 (19.4%)	763
2013	625 (75.7%)	201 (24.3%)	826
2014	278 (79.2%)	73 (20.8%)	351
Total	3659 (81.5%)	830 (18.5%)	4489
χ^2 = 106.6, p<0.001			

Table S2 Discrete time analysis predicting time to next admission by CTO exposure during discrete 12 month follow-up periods with multivariable logistic regression.

Follow-up period (n)	Univariate		Multivariable	
	Odds Ratio (95% CI)	p value	Odds Ratio (95% CI)	p value
0 to 12 months (4489)	1.78 (1.49 to 2.11)	<0.001	1.63 (1.34 to 1.98)	<0.001
12 to 24 months (4489)	1.58 (1.24 to 2.01)	<0.001	1.42 (1.08 to 1/85)	0.011
24 to 36 months (3766)	1.64 (1.19 to 2.26)	0.003	1.53 (1.07 to 2.20)	0.02
36 to 48 months (2995)	2.37 (1.52 to 3.69)	<0.001	2.01 (1.023 to 3.30)	0.006
48 to 60 months (2253)	2.36 (1.36 to 4.09)	0.002	1.84 (1.00 to 3.39)	0.05
Multivariable model adjusted for age, sex, diagnosis, forensic status, antipsychotic route and year of study entry				

Barkhuizen W, *et al. BMJ Open* 2020; 10:e035121. doi: 10.1136/bmjopen-2019-035121

Community Treatment Orders: A controlled electronic case register study
Supplementary Material

Figure S1 Kaplan-Meier survival graph.

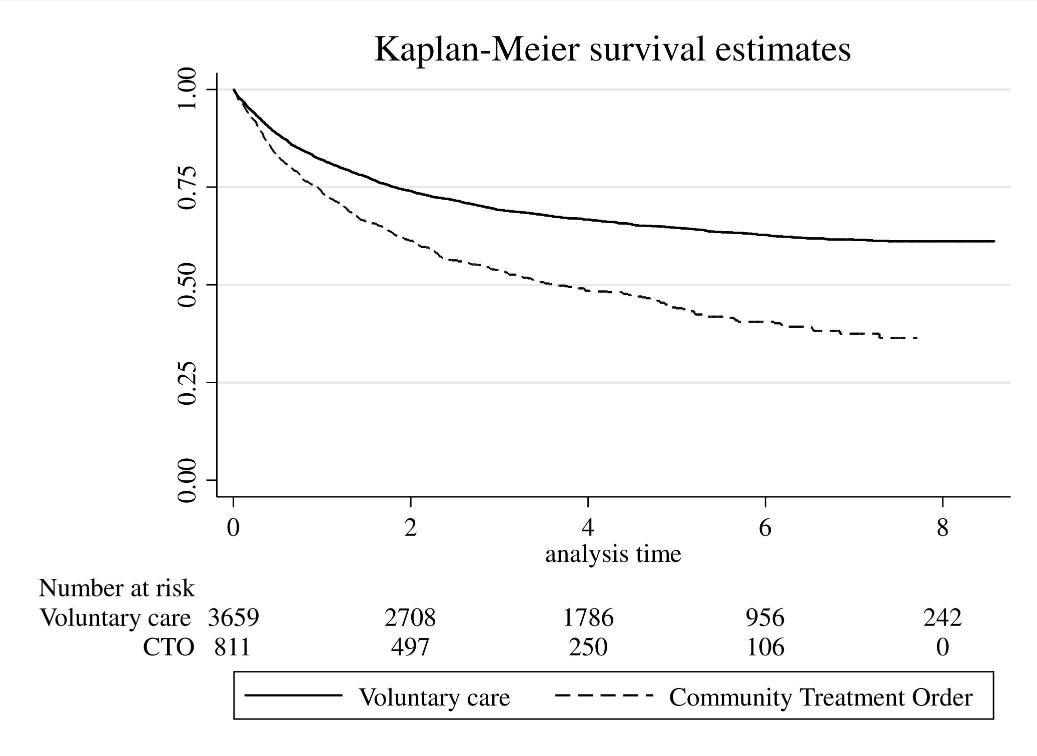

Barkhuizen W, *et al. BMJ Open* 2020; 10:e035121. doi: 10.1136/bmjopen-2019-035121

Community Treatment Orders: A controlled electronic case register study
Supplementary Material

Figure S2 Proportion of individuals with either a reduction or an increase in the amount of days spent in inpatient care in the 24 months before and after index admission, by CTO exposure (n = 4460).

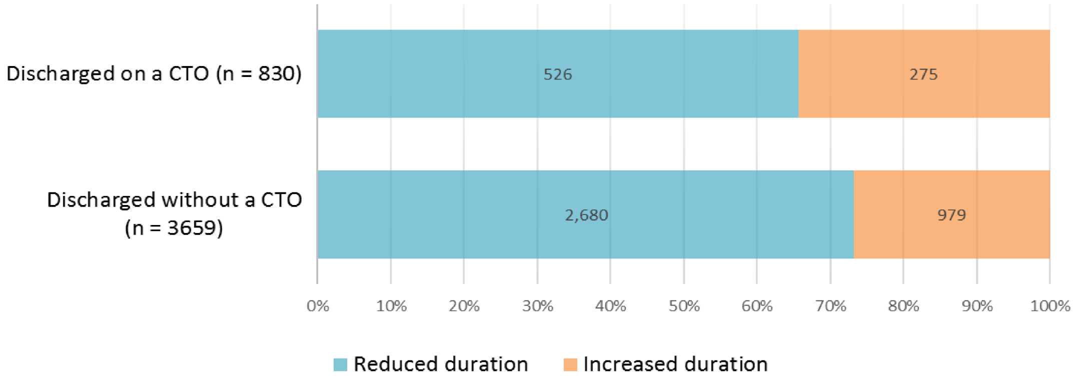

Barkhuizen W, *et al. BMJ Open* 2020; 10:e035121. doi: 10.1136/bmjopen-2019-035121
