# Supplementary material for: Community treatment orders and associations with readmission rates and duration of psychiatric hospital admission: a controlled electronic case register study
Source: BMJ Open. 2020 Mar 5;10(3):e035121. doi: 10.1136/bmjopen-2019-035121 (PMC7059496; doi:10.1136/bmjopen-2019-035121)
Supplement: Author's manuscript [file bmjopen-2019-035121.draft_revisions.pdf]

BMJ Open is committed to open peer review. As part of this commitment we make the peer review history of every article we publish publicly available.

When an article is published we post the peer reviewers' comments and the authors' responses online. We also post the versions of the paper that were used during peer review. These are the versions that the peer review comments apply to.

The versions of the paper that follow are the versions that were submitted during the peer review process. They are not the versions of record or the final published versions. They should not be cited or distributed as the published version of this manuscript.

BMJ Open is an open access journal and the full, final, typeset and author-corrected version of record of the manuscript is available on our site with no access controls, subscription charges or pay-per-view fees (http://bmjopen.bmj.com).

If you have any questions on BMJ Open's open peer review process please email editorial.bmjopen@bmj.com

BMJ Open

Community Treatment Orders and associations with readmission rates and duration of psychiatric hospital admission: A controlled electronic case register study

Journal:	*BMJ Open*
Manuscript ID	bmjopen-2019-035121
Article Type:	Original research
Date Submitted by the Author:	18-Oct-2019
Complete List of Authors:	Barkhuizen, Wikus; King's College London, Department of Psychosis Studies<br>Cullen, Alexis; King's College London, Department of Psychosis Studies<br>Shetty, Hitesh; South London and Maudsley NHS Foundation Trust, Biomedical Research Centre Nucleus<br>Pritchard, Megan; Kings College London, Institute of Psychiatry, Psychology and Neuroscience<br>Stewart, Robert; King's College London, Institute of Psychiatry<br>McGuire, Philip; King's College London, Department of Psychosis Studies<br>Patel, Rashmi; King's College London, Department of Psychosis Studies
Keywords:	MENTAL HEALTH, PSYCHIATRY, EPIDEMIOLOGY, Health policy < HEALTH SERVICES ADMINISTRATION & MANAGEMENT, MEDICAL LAW, Health informatics < BIOTECHNOLOGY & BIOINFORMATICS

SCHOLARONE™
Manuscripts

Community Treatment Orders and associations with readmission rates and duration of psychiatric hospital admission: A controlled electronic case register study

Wikus Barkhuizen [1], Alexis E. Cullen [1], Hitesh Shetty [2], Megan Pritchard [1], Robert Stewart [1,2], Philip McGuire [1] and Rashmi Patel [1]

1. King's College London (Institute of Psychiatry, Psychology and Neuroscience), London, UK
2. South London and Maudsley NHS Foundation Trust, London, UK

Correspondence to:

Rashmi Patel

Tel: +44 (0) 2078480355

E-mail: bmj@rpatel.co.uk

For peer review only

Word count:

Abstract: 293

Total: 4158

For peer review only

Competing interests: All authors have completed the ICMJE uniform disclosure form at www.icmje.org/coi_disclosure.pdf and declare: RS has received research funding from Roche, Janssen, GSK and Takeda. The other authors declare no competing interests.

Contributors: The study was conceived by AEC and RP. Data extraction was performed by WB with support from HS and MP. Statistical analyses and reporting of findings were carried out by WB, supervised by AEC and RP. All authors contributed to study design, manuscript preparation and approved the final version.

Ethics approval: The CRIS data resource received ethical approval as an anonymised dataset for secondary analyses from Oxfordshire REC C (Ref: 08/H0606/71+5).

Source of funding: HS, MP, RS and PM receive funding from the National Institute for Health Research (NIHR) Biomedical Research at South London and Maudsley NHS Foundation Trust and King's College London, which also supports the development and maintenance of the BRC Case Register. RP has received support from a Medical Research Council (MRC) Health Data Research UK Fellowship (MR/S003118/1) and a Starter Grant for Clinical Lecturers (SGL015/1020) supported by the Academy of Medical Sciences, The Wellcome Trust, MRC, British Heart Foundation, Arthritis Research UK, the Royal College of Physicians and Diabetes UK. AEC has received support from a Sir Henry Wellcome Postdoctoral Fellowship (107395/Z/15/Z).

Role of funder: The views expressed are those of the authors and not necessarily those of the NHS, the NIHR or the Department of Health. The funders had no role in the design and conduct of the study; collection, management, analysis, and interpretation of the data;

For peer review only

preparation, review, or approval of the manuscript; and decision to submit the manuscript for publication.

Data sharing: The data accessed by CRIS remain within an NHS firewall and governance is provided by a patient-led oversight committee. Subject to these conditions, data access is encouraged and those interested should contact RS (robert.stewart@kcl.ac.uk), CRIS academic lead.

For peer review only

Abstract

Objectives: Limited evidence is available regarding the effect of Community Treatment Orders (CTOs) on readmission to hospital. We aimed to compare patients placed on CTOs to a control group of patients discharged voluntarily to investigate the association between CTOs and time to readmission and the number of days spent in psychiatric hospital.

Design and setting: An observational study using de-identified electronic health record data from inpatients receiving mental healthcare in South London using the Clinical Record Interactive Search (CRIS) system. Patients discharged from compulsory inpatient treatment between November 2008 and May 2014 were considered to allow for a two-year follow-up period to evaluate outcomes.

Participants: 830 participants discharged on a CTO (mean age 40 years; 63% male) and 3,659 control participants discharged without a CTO (mean age 42 years; 53% male).

For peer review only

Outcome measures: The number of days spent in the community until readmission and the number of days spent in inpatient care in the two years prior to and the two years following the index admission.

Results: The mean duration of a CTO was 3.2 years. Patients receiving care from forensic psychiatry services were 5 times more likely and patients receiving a long-acting injectable antipsychotic were twice as likely to be placed on a CTO. There was a significant association between CTO receipt and readmission in adjusted models (hazard ratio: 1.60, 95% CI 1.42 to 1.80, p<0.001). Compared to controls, patients on a CTO spent 17.3 additional days (95% CI 4.0 to 30.6, p=0.011) in a psychiatric hospital in the 2 years following index admission.

Conclusions: Many patients spent longer on CTOs than initially anticipated by policy makers. Those on CTOs are readmitted sooner and spend more time in hospital. These findings merit consideration in future amendments to the UK Mental Health Act.

Strengths and limitations of this study

For peer review only

This large observational study of CTOs is the first in England and Wales to include an appropriate control group to allow for robust evaluation of outcomes.

The use of clinical records allowed for evaluation of CTOs in real-world clinical practice, an advantage when it comes to generalisability of the findings.

The design of this study limited the risk of follow-up bias since all patients within the catchment area could be included in the analyses.

While we were able to account for several factors associated with the receipt of CTOs, we were not able to account for differences in illness severity between CTO and control patients.

For peer review only

Introduction

Introduced in England and Wales under the 2007 Mental Health Act (MHA), Community Treatment Orders (CTOs) allow for compulsory clinical monitoring of people with serious mental disorders within community care settings and recall to inpatient care for a 72-hour assessment period. Where longer inpatient treatment is deemed most appropriate a CTO can be revoked, in which case the existing section under which the patient was initially hospitalised (if still in place) takes effect. Only patients already hospitalised involuntarily for treatment of a mental disorder are eligible for a CTO. Orders require renewal every 6 months, and, if not extended, lapse at which point the patient is effectively discharged from compulsory treatment. Despite initial scepticism from mental health practitioners,[1] their use has exceeded initial government projections[2] with about 5,000 currently being issued per year.[3]

Evidence from England and Wales on the effectiveness of CTOs is limited: The only randomised controlled trial (RCT) found no evidence that CTOs reduce readmission rates but excluded forensic patients, thereby limiting generalisability.[4] Most observational studies conducted in England and Wales found support for their

For peer review only

use in reducing readmission rates and days spent in hospital.[5-7] However, these studies were small and underpowered, examined outcomes within the first year only, and failed to include a control group. As such, the effects of CTOs on readmission rates and duration of readmission episodes are likely to have been overestimated. A recent meta-analysis of CTO studies conducted internationally concluded that studies without a control condition found evidence for a reduction in readmission rates and bed-days whilst those with a control condition did not.[8] However, due to differences between jurisdictions in health care systems and compulsory community treatment programmes and legislation, results may not be generalisable to England and Wales.

Aims

We sought to gather data from a large catchment area in South London using the electronic mental healthcare records for all patients who were involuntarily hospitalised since the introduction of CTOs in 2008. We aimed to provide the first robust estimate of the effect of CTOs on relapse rates (using hospital readmission as a proxy) by including an appropriate control group of those discharged to voluntary

For peer review only

community care whilst adjusting for the demographic and clinical features associated with CTO receipt. Second, we aimed to compare patients discharged on CTOs to those discharged voluntarily on the number of days spent in inpatient care in the two years following the index discharge.

Methods

Study population and data collection

Data were obtained from anonymised electronic health records of mental health services provided by the South London and Maudsley National Health Service (NHS) Foundation Trust (SLaM). Serving a catchment of 1.3 million residents in the boroughs of Lambeth, Southwark, Lewisham and Croydon, SLaM provides more than 230 services that include specialist psychosis services, inpatient wards, community and outpatient services. SLaM treats an estimated 5,300 inpatients and 45,000 outpatients per year. The Clinical Record Interactive Search (CRIS) system[9] [10] collates de-identified patient data from SLaM and contains both structured and free text fields of routine clinical data including case notes and correspondence.[11]

For peer review only

The analysed sample consisted of all SLaM patients who received and completed an involuntary inpatient episode under Section 3 or Section 37 of the MHA between November 2008 and May 2014, allowing for a follow-up period in all cases by using data up to July 2016.

The authors assert that all procedures contributing to this work comply with the ethical standards of the relevant national and institutional committees on human experimentation and with the Helsinki Declaration of 1975, as revised in 2008. All procedures involving human subjects/patients were approved by the CRIS Oversight Committee which is responsible for ensuring all secondary research applications comply with legal requirements and ethical approval obtained from the Oxfordshire Research Ethics Committee C (reference 18/SC/0372).

Measures

Exposure to a community treatment order

The exposure measure was whether patients discharged from an involuntary psychiatric hospital admission under Section 3 or Section 37 of the MHA were placed on a CTO or discharged without a CTO. CTO status and psychiatric hospital

For peer review only

admission data were gathered from structured fields within the health record dataset. A manual free-text search on 50 random cases was conducted to confirm that no CTO orders were missed using data from these structured fields. 471 (12.9%) control patients and 140 (16.9%) CTO patients were discharged from more than one involuntary psychiatric hospital admission during the study period. 359 patients in the CTO group (43.3%) had been placed on a CTO more than once. For patients who had never been placed on a CTO but had more than one involuntary admission under Section 3 or Section 37 of the MHA, the most recent Section 3 or 37 discharge date was taken as the index date. Where a patient had more than one CTO the most recent discharge to CTO was taken as the index date.

Covariates

Demographic and clinical information was gathered to identify factors associated with CTO receipt. Sex, ethnicity, age at the time of the index admission, the mode of involuntary admission under which patients were admitted (either Section 3 issued by an approved mental health practitioner (AMHP) and two doctors or Section 37 issued by criminal courts) and forensic status (determined if individuals were

For peer review only

discharged from a forensic ward or to a forensic service) were collected from structured fields. Collected data included the start and end date of the index admission and the duration of the index admission in days. Psychiatric diagnosis was obtained from both structured fields on CRIS and free text searches to improve the reliability of information at the time of the index episode. Diagnoses were subsequently grouped according to ICD-10 codes.[12]

We extracted the three most recent antipsychotic medications prescribed prior to discharge and method of administration from structured fields. A variable indexing antipsychotic type (oral, long-acting injectable [LAI], or none) was created. Those on more than one antipsychotic were classified in the LAI group if any of the most recently prescribed drugs two weeks prior to discharge were administered as LAI (to take into account combined oral and LAI treatment during initiation of LAI). We chose to distinguish between oral and LAI antipsychotics as LAI antipsychotics are associated with different rates of psychiatric hospital readmission compared to oral antipsychotics.

We included the year of study entry as a covariate as a measure of changes in mental healthcare service provision over time which may have been independently associated with risk of psychiatric hospital admission and number of inpatient days.

Outcomes of Community Treatment Orders

The primary outcome measure was the number of days spent in the community from the discharge date of the index hospital admission until readmission to a psychiatric ward or the end of the follow-up period (31st July 2016). We extracted data on mortality and censored individuals who died during the follow-up period in survival analyses. We additionally measured the number of days spent in inpatient care in the two years prior to and the two years following the index admission.

Missing Data

There were no missing data for predictor (CTO status) or outcome (readmission/number of inpatient days) variables. The number of patients with missing data for each covariate entered into multivariable analyses was as follows: age: 41; sex: 0; diagnosis: 43; forensic status: 198; antipsychotic route: 107; year of study entry: 0; number of inpatient days in 2 years prior to index admission: 0. Where

For peer review only

patients had missing covariate data, they were dropped from multivariable analyses. 92.3% of patients had complete covariate data.

Analyses

All analyses were conducted in Stata 13[13] and statistical significance was defined at $p<0.05$. Univariate logistic regression models were first performed to assess the association between CTO status and demographic/clinical factors (sex, age, ethnicity, diagnosis, forensic status, the mode of admission and the route of administration of antipsychotic medication). A fully adjusted model using multivariable logistic regression was subsequently performed with all covariates entered simultaneously to predict CTO status.

To adjust for censoring based on the study follow-up period, mean and median CTO duration were estimated using the “stci” and “stsum” commands in STATA censored on 31st July 2016 (the end of the study follow-up window).

Kaplan-Meier curves and log-rank tests were used to assess differences in the observed survival rates (time to next admission) between CTO and voluntarily discharged patients. To assess the association between CTO exposure and time to

For peer review only

next admission, unadjusted Cox regression models were performed. Covariates that were significantly associated with CTO exposure in the adjusted logistic regression analyses were then added to the Cox regression in a multivariable model. Overall significance of the Cox regression model was assessed using the −2 log-likelihood ratio test. A Schoenfeld residuals test and visual inspection of Nelson-Aalen cumulative hazard curves were used to test the proportional-hazards assumption. An additional discrete time analysis was performed where the proportional-hazards assumption was not met.

The CTO and control group were compared on the number of days spent in a psychiatric hospital two years after the index date using multiple linear regression with the same covariates as used in the multivariable Cox regression on time to readmission plus an additional covariate measuring the number of days spent in inpatient care two years prior to the index admission as this factor was associated with increased number of days spent in a psychiatric hospital subsequently (Pearson coefficient: 0.23, $p<0.001$).

For peer review only

Results

The cohort comprised 4,489 SLaM patients discharged from involuntary inpatient care between 2008 and 2014. Of these, 830 (18.5%) were placed on a CTO at least once while the remaining 3659 (81.5%) were not. The mean age at discharge of those on CTOs was 39.5 years (s.d. = 13.3) and those discharged without a CTO 42.1 years (s.d. = 16.4). The CTO group had a larger proportion of males compared to those not on a CTO (62.9% v 52.8%). Table 1 summarises the demographic details of the sample by CTO exposure.

The mean duration of CTO was 3.20 years (95% CI 2.95 to 3.45). The median duration of CTO was 2.65 years (IQR 0.50 to 5.65). Of those whose CTOs ended before the end of the study period (n = 434), 19.0% were discharged, 15.7% had their CTOs revoked (i.e., they remained in hospital under their previous MHA Section), and CTOs lapsed (i.e., they were not renewed at 6 months) for 12.4% of patients.

489 patients died during the study period. Of these, 432 were in the control group and 57 were in the CTO group. The overall mortality rate was 2.14% per year.

For peer review only

The mortality rate for the control group was 2.27% per year. The mortality rate for the CTO group was 1.53% per year (hazard ratio compared to controls 0.66, 95% CI 0.50 to 0.88).

A breakdown of year of study entry is provided in the online supplementary table S1 and indicates that rates of CTO usage have varied over time with peak usage occurring in 2013.

Predictors of receipt of a CTO

In unadjusted logistic regression models (table 1), several significant predictors of CTO exposure were identified. Females had lower odds than males of being placed on a CTO. Those between 25-35 years of age more likely to receive a CTO, black patients had higher odds of receiving a CTO compared to white patients. Compared to receiving a diagnosis of schizophrenia, those with other diagnoses had reduced odds of being placed on a CTO. The odds of receiving a CTO were five times higher among those receiving care from forensic services compared to those in secondary (non-forensic) care and 2.3 times higher in those on LAI relative to those on oral antipsychotics. Patients on a Section 37 (typically issued by criminal courts) during

For peer review only

their index inpatient episode had twice the odds of being placed under a CTO compared to patients on a Section 3.

A logistic regression model that adjusted for all predictors (table 1; $\chi^2(15) = 320.58$, $p < 0.001$; pseudo-$R^2 = 0.08$) indicated that all factors reported above, with the exception of patient ethnicity, remained significantly associated with CTOs, albeit with attenuated odds ratios.

Time to readmission

The overall mean time to re-admission was 5.55 years (95% CI 5.44 to 5.66). The mean time to re-admission for the control group was 5.82 years (95% CI 5.70 to 5.94). The mean time to re-admission for the CTO group was 4.02 years (95% CI 3.80 to 5.25). Kaplan-Meier curves (see online supplementary figure S1) comparing the observed survival time to next admission indicated that patients on a CTO were likely to be readmitted sooner than patients who were not on a CTO (univariate hazard ratio: 1.76, 95% CI 1.58 to 1.96, p<0.001). A multivariable Cox regression model (table 2) confirmed the association between CTO exposure and reduced time to readmission which remained significant after adjusting for covariates. Figure 1

For peer review only

illustrates the survival rates depending on CTO status. The Schoenfeld residuals test indicated that the proportional hazards assumption was not met (χ^2 =14.3, p<0.001) suggesting that the effect of CTO exposure on time next admission was not constant over time. We therefore conducted a discrete time analysis using multivariable logistic regression on discrete periods of 12 months of follow-up (online supplementary table S2) that confirmed the association between CTO receipt and reduced time to next readmission across different periods of follow-up.

Length of stay

Patients not on a CTO spent a mean of 148.3 days (std dev: 164.1) and median of 85 days (IQR 43 to 183) in a psychiatric hospital in the two years after the index date. Patients who were on a CTO spent a mean of 177.7 days (std dev: 178.4) and median of 116.5 days (IQR 52 to 253) in a psychiatric hospital in the two years after the index date. After adjusting for covariates in multiple linear regression (table 3), Those on CTOs spent 17.3 additional days (95% CI 4.0 to 30.6) in a psychiatric hospital compared to patients not on a CTO.

For peer review only

Online supplementary figure S2 illustrates that a greater proportion of patients who were not placed on a CTO spent less time in hospital compared to prior to their index admission than patients who were placed on a CTO.

For peer review only

Discussion

Main findings

To the best of our knowledge, this is the largest observational study to evaluate the predictors and outcomes of CTOs in England and Wales. Importantly, the inclusion of a control group of patients discharged to voluntary care allowed for a robust evaluation of CTOs in real clinical settings. Those placed on CTOs were more likely to be male, in the youngest (under the age of 25) and oldest (over 55) age groups, have a diagnosis of schizophrenia-spectrum or depressive disorders, have been treated in forensic mental health services, and in receipt of LAI antipsychotics. The greatest proportion of admissions resulting in discharge to a CTO occurred in 2013. CTOs were associated with a higher rate of readmission, even after accounting for associated demographic and clinical features. Patients on a CTO had a greater number of days spent in psychiatric hospital in the 2 years following discharge from index admission than patients not on a CTO.

Community Treatment Orders and relapse rates

For peer review only - http://bmjopen.bmj.com/site/about/guidelines.xhtml

We found that, at any given time during the follow-up period, those on CTOs had an increased risk of being readmitted to psychiatric inpatient treatment compared to those voluntarily discharged after controlling for factors associated with CTO status. Previous studies in England and Wales[5-7] reported a reduction in hospital days and admission rates after patients were placed on a CTO compared to the two years prior to the order. These studies employed uncontrolled before-and-after designs on samples ranging from 20 to 37 patients, all recruited from a single service. Consequently, both statistical power and the generalisability of findings were limited. Also impacting on the generalisability, one study[5] excluded those on short duration CTOs. In the absence of control conditions, effects other than CTO exposure, including the natural trajectory of disease and other treatment effects, are more likely to have accounted for some of the improvement in the rates and duration of readmissions previously reported.

There are several possible explanations for why those on CTOs had higher rates of psychiatric hospital admission compared to controls in our study. One plausible explanation is that patients with more severe symptoms or a history of relapse were more likely to receive CTOs. Hospitalisation rates may also be higher

For peer review only

due to the relative ease of readmission of deteriorating patients under the CTO pathway, which does not require a Mental Health Act assessment to be conducted. An alternative possibility is that CTOs were not effective at reducing relapse rates. Whilst we controlled for factors associated with receipt of CTOs (forensic status, diagnosis and the route of antipsychotic administration), we were not able to account for possible differences in illness severity between CTO patients and controls, nor did we compare relapse rates prior to and after being placed on CTOs.

Our study also examined CTO outcomes in terms of differences in the number of days spent in psychiatric hospitals. After adjusting for covariates including the amount of time spent in a psychiatric hospital prior to index admission, those on CTOs spent 17 additional days in a psychiatric hospital during the follow-up period compared to patients who were not placed on a CTO. Our findings are in line with previous studies that have examined the number of days spent in hospital pre- and post-CTO exposure.[5-7]; however, these studies failed to include a control group.

The duration of Community Treatment Orders

For peer review only

We report that, once a CTO is issued, many patients are kept under this treatment option for extensive periods. The mean duration of a CTO in our study was 3 years, far exceeding initial government projections of 9 months.[14] This is informative because while official figures on the number of CTOs issued per year are readily available,[3] national statistics on their duration are hard to come by. In the current sample, CTOs also lasted longer than previously found in samples from England and Wales. For instance, we found that CTOs lasted three times as long compared to Dye et al[7] who reported an average duration of 52.6 weeks (s.d. = 31.7). A likely reason for this may be the longer follow-up period in this study; At the end of Dye et al's 2-year follow-up period, 35% of their sample were still on CTOs. That a large proportion of the eligible patient population are exposed to compulsory community treatment, often for long periods of time, underscores the importance of understanding the characteristics of and outcomes for those on CTOs.

Demographic and clinical features of patients who receive Community Treatment Orders

For peer review only

The age and sex distributions within the CTO subsample were similar to previous reports.[6 7 15] Unlike previous studies, we were able to compare the demographics of those on CTOs to those discharged without a CTO: Taking into account other factors, those already hospitalised under the MHA were 4.4 times more likely to receive a CTO if they were in forensic services, twice as likely to be on LAI than oral antipsychotics, more likely male and with a diagnosis of a schizophrenia-spectrum disorder compared to bipolar, other psychotic or miscellaneous disorders (but not depression).

Almost 20% of those placed on CTOs were either hospitalized within forensic wards or discharged to forensic services. Consequently, those with forensic service involvement were more likely than non-forensic patients to be subject to compulsory treatment. Considering that the most commonly cited reasons why patients are placed on CTOs in England and Wales relates to clinical considerations such as improving adherence to treatment,[16] as opposed to monitoring risk, this result is somewhat surprising. It is possible that CTOs may have been used primarily for protective or monitoring reasons among forensic patients more commonly than previously suggested. In the absence of further investigation into the motivations for

For peer review only - http://bmjopen.bmj.com/site/about/guidelines.xhtml

issuing CTOs and their conditions within this sample, it is not clear what underlies the reason that forensic status was found to be predictive of being placed on a CTO.

The proportion of patients of black ethnicity being placed on CTOs was similarly high to that reported by Patel et al[15] in their cohort within the same geographic area of London. Patel et al. speculated that their finding may have been due to the high number of black patients sectioned under the MHA. The current study was able to explore this phenomenon in further detail as it included all those detained under the MHA and found that black patients who were already in psychiatric hospitals were significantly more likely to be placed on a CTO than other patients. However, the association between ethnicity and CTO exposure did not remain significant after considering other predictors of being started on a CTO, including forensic, clinical and demographic characteristics, suggesting that these factors explained the positive association we observed between black ethnicity and CTO receipt.

Like others, this study found that a large proportion of those on CTOs were prescribed LAI formulation antipsychotics (41%).[5 6 15 17] Moreover, this study

For peer review only

demonstrated that those on a CTO had twice the odds of being prescribed LAIs than those not on a CTO, a finding that remained significant after controlling for the effect of diagnosis. Several reasons for a preference by psychiatrists for prescribing LAI formulation antipsychotics to those on CTOs have been proposed: Patel et al[15] suggested that those less likely to adhere are placed on LAI and therefore at greater risk of relapse. This would make them likely candidates for a CTO. Adherence to LAIs is also easier to monitor and therefore preferable for reasons of enforcement.

Strengths and limitations

The advantage of using health records to investigate the use and effects of CTOs is that they provide data which represents real-world clinical practice which may be more generalisable than findings from RCTs whose participants may not be representative of the wider population receiving mental healthcare. Additionally, follow-up bias is limited as most subjects are included at all time points of data gathering unless they leave the area or die.[18 19] The SLaM BRC CRIS system has specific advantages over many other electronic case registers: with access to over

For peer review only

400,000 patient records and combining structured fields and free-text searches from fully digitised case registers, it has both the quality of smaller databases and the quantity of data only large registers provide.[9] On the other hand, the use of routine clinical data limits the possible number of covariates that can be reliably controlled for and does not rule out confounding by indication. For instance, this study did not control for illness severity. Electronic patient records are also susceptible to erroneous or partial recording of treatment events. During the study period patients may have moved out of the catchment area or into the catchment area from elsewhere. As the data source only included records from SLaM, CTO and hospital admission episodes in other mental healthcare providers would not have been included.

Using the most recent CTO episode that patients were subject to (in cases where the same patient was subject to more than one CTO during the window period) enabled the study to report on current practices; the way in which CTOs have been used in other jurisdictions has indicated possible change over time as practitioners become more familiar with the legislation.[20]

For peer review only

The rates of readmission reported here should be interpreted with some cautions as our survival analysis did not meet the assumption of proportional hazards. However, discrete time analysis indicated that the association of CTOs with increased rates of readmission persisted across all periods of follow-up.

Implications

There are several implications for both current and future research into the effects of CTOs. We found that patients spend several years on a CTO, far longer than the original projected duration of 9 months. At the same time, our study demonstrated that patients on CTOs have greater rates of readmission and spend longer in psychiatric hospital than patients who are not on CTOs, going against previously published studies which did not include a control group.

We found that certain demographic and clinical features, such as ethnicity and forensic background, are strongly associated with CTO exposure draws into question the generalisability of findings reported elsewhere, including those from the OCTET trial that excluded forensic patients. Several observations on the use of CTOs

For peer review only

warrant further investigation, including the influence of LAIs on outcomes, and the effects of extensive periods of community compulsion on patient engagement.

We found a higher incidence of CTOs among black patients adding to previous findings which indicate that black and minority ethnic (BME) groups are overrepresented in compulsory mental healthcare admission and treatment.

In conclusion, our study demonstrates that the potential benefits and harms of CTOs remain unclear. Patients may spend several years on a CTO and they are not associated with a reduction in psychiatric hospital admission or with less time spent in a psychiatric hospital. The implementation of CTOs in future amendments of the UK Mental Health Act should be reviewed in light of these findings.[21]

For peer review only

Author details

Wikus Barkhuizen (MSc), Postgraduate student, King's College London (Institute of Psychiatry, Psychology and Neuroscience), London, UK

Alexis Cullen (PhD), Sir Henry Wellcome Postdoctoral Fellow, King's College London (Institute of Psychiatry, Psychology and Neuroscience), London, UK

Hitesh Shetty (MSc), Clinical Informatician, Maudsley Biomedical Research Centre, South London and Maudsley NHS Foundation Trust, London, UK

Megan Pritchard (MSc), Training and Development Lead, Maudsley Biomedical Research Centre, South London and Maudsley NHS Foundation Trust, London, UK

Robert Stewart (MD), Professor of Psychiatric Epidemiology & Clinical Informatics, King's College London (Institute of Psychiatry, Psychology and Neuroscience), London, UK

Philip McGuire (MD PhD), Professor of Psychiatry & Cognitive Neuroscience, King's College London (Institute of Psychiatry, Psychology and Neuroscience), London, UK

For peer review only

Rashmi Patel (BM BCh PhD), MRC Health Data Research UK Fellow, King's College London (Institute of Psychiatry, Psychology and Neuroscience), London, UK

For peer review only

References

1. Churchill R. International experiences of using community treatment orders (PhD diss.). Kings College London, 2007.
2. Trevithick L, Carlile J, Nodiyal S, *et al*. Community treatment orders: an analysis of the first five years of use in England. *Br J Psychiatry* 2018;**212**:175-79.doi: 10.1192/bjp.2017.51
3. NHS Digital. Mental Health Act Statistics, Annual Figures: 2016-17, Experimental statistics, 2017. https://www.gov.uk/government/publications/independent-review-of-the-mental-health-act-interim-report (Accessed 8 Jul 2019).
4. Burns T, Rugkasa J, Molodynski A, *et al*. Community treatment orders for patients with psychosis (OCTET): a randomised controlled trial. *Lancet* 2013;**381**:1627-33.doi: 10.1016/s0140-6736(13)60107-5
5. Rawala M, Gupta S. Use of community treatment orders in an inner-London assertive outreach service. *Psychiatr Bull* 2014;**38**:13-8.doi: 10.1192/pb.bp.112.042184
6. Awara MA, Jaffar K, Roberts P. Effectiveness of the Community Treatment Order in streamlining psychiatric services. *J Ment Health* 2013;**22**:191-7.doi: 10.3109/09638237.2013.775408
7. Dye S, Dannaram S, Loynes B, *et al*. Supervised community treatment: 2-year follow-up study in Suffolk. *Psychiatrist Online* 2012;**36**:298-302.doi: 10.1192/pb.bp.111.036657
8. Barnett P, Matthews H, Lloyd-Evans B, *et al*. Compulsory community treatment to reduce readmission to hospital and increase engagement with community care in people with mental illness: a systematic review and meta-analysis. *Lancet Psychiatry* 2018;**5**:1013-22.doi: 10.1016/s2215-0366(18)30382-1

For peer review only

9. Perera G, Broadbent M, Callard F, *et al*. Cohort profile of the South London and Maudsley NHS Foundation Trust Biomedical Research Centre (SLaM BRC) Case Register: current status and recent enhancement of an Electronic Mental Health Record-derived data resource. *BMJ Open* 2016;**6**:e008721.doi: 10.1136/bmjopen-2015-008721

10. Stewart R, Soremekun M, Perera G, *et al*. The South London and Maudsley NHS Foundation Trust Biomedical Research Centre (SLAM BRC) case register: development and descriptive data. *BMC Psychiatry* 2009;**9**:51.doi: 10.1186/1471-244x-9-51

11. Fernandes AC, Cloete D, Broadbent MT, *et al*. Development and evaluation of a de-identification procedure for a case register sourced from mental health electronic records. *BMC Med Inform Decis Mak* 2013;**13**:71.doi: 10.1186/1472-6947-13-71

12. World Health Organization. The ICD-10 classification of mental and behavioural disorders: diagnostic criteria for research. Geneva: World Health Organization 1993.

13. StataCorp. Stata Statistical Software: Release 13. College Station, TX: StataCorp LP. 2013.

14. Department of Health. Mental Health Bill: Regulatory Impact Assessment, 2006. https://webarchive.nationalarchives.gov.uk/20081212000105/http://www.dh.gov.uk/en/Publicationsandstatistics/Legislation/Regulatoryimpactassessment/DH_076477 (Accessed 22 Dec 2018).

15. Patel MX, Matonhodze J, Baig MK, *et al*. Naturalistic outcomes of community treatment orders: antipsychotic long-acting injections versus oral medication. *J Psychopharmacol* 2013;**27**:629-37.doi: 10.1177/0269881113486717

16. DeRidder R, Molodynski A, Manning C, *et al*. Community treatment orders in the UK 5 years on: a repeat national survey of psychiatrists. *BJPsych Bull* 2016;**40**:119-23.doi: 10.1192/pb.bp.115.050773

For peer review only

17. Rugkasa J, Molodynski A, Yeeles K, *et al*. Community treatment orders: clinical and social outcomes, and a subgroup analysis from the OCTET RCT. *Acta Psychiatr Scand* 2015;**131**:321-9.doi: 10.1111/acps.12373

18. Kisely S, Campbell LA, Preston NJ, *et al*. Can epidemiological studies assist in the evaluation of community treatment orders? - The experience of Western Australia and Nova Scotia. *Int J Law Psychiatry* 2006;**29**:507-15. doi: 10.1016/j.ijlp.2006.01.006

19. Swanson JW, Swartz MS. Why the evidence for outpatient commitment is good enough. *Psychiatr Serv* 2014;**65**:808-11.doi: 10.1176/appi.ps.201300424

20. Kisely S, Preston N, Xiao J, et al. An eleven-year evaluation of the effect of community treatment orders on changes in mental health service use. *J Psychiatr Res* 2013;**47**:650-6.doi: 10.1016/j.jpsychires.2013.01.010

21. Department of Health and Social Care. Independent review of the Mental Health Act: interim report, 2018. https://www.gov.uk/government/publications/independent-review-of-the-mental-health-act-interim-report (Accessed 19 Nov 2018).

For peer review only

Tables and Figure legends

Table 1 Sample characteristics and logistic regression of factors associated with Community Treatment Orders

	Total group (*N* = 4489)		Controls (*n* = 3659)		CTO (*n* = 830)		Unadjusted models			Adjusted models		
	n	**%**	***n***	**%**	***n***	**%**	**OR**	***CI***	***p***	**OR**	***CI***	***p***
**Sex**												
Male	2,453	54.6	1,931	52.8	522	62.9	*Ref*	-	-	-	-	-
Female	2,036	45.4	1,728	47.2	308	37.1	0.66	*0.56-0.77*	<.001	0.83	*0.71-0.99*	0.042
**Age group**												
<25	683	15.4	577	15.8	106	13.4	0.69	*0.54-0.89*	0.005	0.76	*0.57-1.01*	0.063
25-34	1045	23.5	826	22.6	219	27.8	*Ref*	-	-	-	-	-
35-44	1,027	23.1	825	22.6	202	25.6	0.92	*0.75-1.14*	0.466	0.91	*0.72-1.15*	0.415
45-55	880	19.8	700	19.1	180	22.8	0.97	*0.78-1.21*	0.786	0.94	*0.74-1.20*	0.642
> 55	813	18.3	731	20.0	82	10.4	0.42	*0.32-0.56*	<.001	0.48	*0.36-0.65*	<.001
**Ethnicity**												
White	1,728	38.5	1,449	39.6	279	33.6	*Ref*	-	-	-	-	-
Black	2,192	48.9	1,727	47.2	465	56.0	1.40	*1.19-1.65*	<.001	1.08	*0.89-1.30*	0.457
Other	566	12.6	480	13.1	86	10.4	0.93	*0.72-1.21*	0.591	0.79	*0.59-1.07*	0.134
**Primary diagnosis**												
Schizophrenia	2092	47.1	1,609	44.0	483	61.2	*Ref*	-	-	-	-	-
Other psychotic	628	14.1	544	14.9	84	10.7	0.51	*0.40-0.66*	<.001	0.60	*0.46-0.79*	<.001
Bipolar and related	630	14.2	554	15.2	76	9.6	0.46	*0.35-0.59*	<.001	0.59	*0.44-0.78*	<.001
Depressive disorders	428	9.6	365	10.0	63	8.0	0.57	*0.43-0.77*	<.001	0.93	*0.68-1.26*	0.629
Other	668	15.0	585	16.0	83	10.5	0.47	*0.37-0.61*	<.001	0.63	*0.47-0.84*	0.002
**Forensic status**												
Secondary care	3983	92.8	3318	95.6	665	81.1	*Ref*	-	-	-	-	-
Forensic patient	308	7.2	153	4.4	155	18.9	5.05	*3.98-6.42*	<.001	4.41	*3.40-5.72*	<.001
**Route of antipsychotic**												
Oral	3162	72.2	2741	74.9	421	58.2	*Ref*	-	-	-	-	-
LAI	1,142	26.1	844	23.1	298	41.2	2.30	*1.94-2.72*	<.001	2.18	*1.83-2.61*	<.001
None	78	1.8	74	2.0	4	0.6	0.35	*0.13-0.97*	0.043	0.53	*0.18-1.50*	0.23
**Mode of admission**												
Section 3	4284	96.3	3546	96.9	738	93.5	*Ref*	-	-	-	-	-
Section 37	164	3.7	113	3.1	51	6.5	2.17	*1.54-3.05*	<.001	1.42	*0.93-2.16*	0.105

Note: CTO = Community Treatment Orders; LAI = Long-acting injectable; Adjusted models accounting for all predictor variables entered simultaneously into multiple logistic models.

For peer review only

Table 2 Multivariable Cox regression analysis of time to next admission following discharge from index admission (*n* = 4,144)

Factor	Hazard Ratio (95% CI)	p value
**Control group (reference)**		
Community Treatment Order (CTO) group	1.60 (1.42 to 1.80)	<0.001
**Age (reference: <25 years)**		
25-34 years	1.02 (0.87 to 1.18)	0.84
35-44 years	0.89 (0.76 to 1.04)	0.13
45-55 years	0.80 (0.68 to 0.94)	0.007
> 55 years	0.65 (0.54 to 0.78)	<0.001
**Sex (reference: Male)**		
Female	0.95 (0.86 to 1.04)	0.27
**Diagnosis (reference: Schizophrenia)**		
Other psychotic disorder	0.90 (0.78 to 1.05)	0.18
Bipolar Disorder	1.23 (1.07 to 1.41)	0.004
Depressive disorders	1.19 (1.01 to 1.41)	0.041
Other	0.81 (0.68 to 0.95)	0.011
**Forensic status (reference: Not forensic)**		
In forensic services	1.12 (0.94 to 1.34)	0.22
**Antipsychotic route (reference: Oral)**		
Long-acting injectable (LAI/depot)	1.11 (0.99 to 1.23)	0.063
None	0.47 (0.25 to 0.85)	0.013
**Year of study entry (reference: 2008)**		
2009	1.20 (0.98 to 1.46)	0.083
2010	1.29 (1.06 to 1.58)	0.013
2011	1.47 (1.21 to 1.79)	<0.001
2012	1.81 (1.49 to 2.19)	<0.001
2013	2.02 (1.66 to 2.45)	<0.001
2014	2.45 (1.96 to 3.08)	<0.001
Analysis adjusted for all variables reported in this table		

For peer review only

Table 3 Multiple linear regression investigating number of inpatient days in a psychiatric hospital during the 2 years following discharge from index admission (*n* = 4,190)

Factor	β Coefficient (95% CI)	p value
**Control group (reference)**		
Community Treatment Order (CTO) group	17.3 days (4.0 to 30.6)	0.011
**Age (reference: <25 years)**		
25-34 years	-6.5 days (-22.4 to 9.5)	0.43
35-44 years	-23.7 days (-39.7 to -7.7)	0.004
45-55 years	-12.6 days (-29.2 to 4.0)	0.14
> 55 years	13.9 days (-2.9 to 30.8)	0.11
**Sex (reference: Male)**		
Female	-17.4 days (-27.2 to -7.7)	<0.001
**Diagnosis (reference: Schizophrenia)**		
Other psychotic disorder	-21.1 days (-35.5 to -6.7)	0.004
Bipolar Disorder	-40.2 days (-54.6 to -25.8)	<0.001
Depressive disorders	-33.6 days (-50.5 to -16.7)	<0.001
Other	28.7 days (13.7 to 43.8)	<0.001
**Forensic status (reference: Not forensic)**		
In forensic services	33.4 days (14.1 to 52.7)	0.001
**Antipsychotic route (reference: Oral)**		
Long-acting injectable (LAI/depot)	-15.8 days (-26.8 to -4.8)	0.005
None	29.0 days (-9.0 to 67.0)	0.14
**Year of study entry (reference: 2008)**		
2009	-13.0 days (-31.4 to 5.4)	0.17
2010	-9.2 days (-27.6 to 9.2)	0.33
2011	-12.6 days (-30.7 to 5.4)	0.17
2012	-17.6 days (-35.1 to -0.1)	0.049
2013	-3.8 days (-21.2 to 13.6)	0.67
2014	-3.5 days (-24.9 to 18.0)	0.75
Number of inpatient days in 2 years prior to start of index admission	0.31 days (0.27 to 0.35)	<0.001
Analysis adjusted for all variables reported in this table		

For peer review only

Figure 1 Graph of cox survival function comparing time to next admission between CTO and controls (*n* = 4,439)

<Please see additional file>

For peer review only

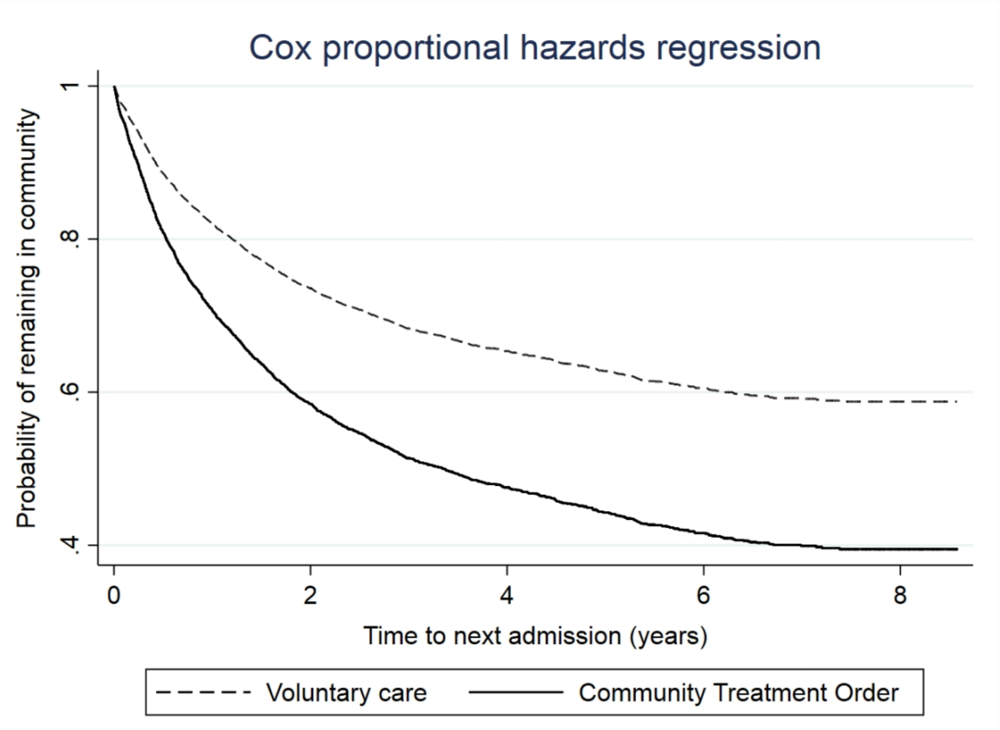

Graph of cox survival function comparing time to next admission between CTO and controls (n = 4,439)

Community Treatment Orders: A controlled electronic case register study
Supplementary Material

Table S1 Number of patients in CTO and control group by year of study entry (*n* = 4,489)

**Year of study entry**	**Control group n (%)**	**CTO group n (%)**	**Total**
2008	560 (96.2%)	22 (3.8%)	582
2009	522 (81.3%)	120 (18.7%)	642
2010	503 (79.2%)	132 (20.8%)	635
2011	556 (80.6%)	134 (19.4%)	690
2012	615 (80.6%)	148 (19.4%)	763
2013	625 (75.7%)	201 (24.3%)	826
2014	278 (79.2%)	73 (20.8%)	351
Total	3659 (81.5%)	830 (18.5%)	4489
χ^2 = 106.6, p<0.001			

Table S2 Discrete time analysis predicting time to next admission by CTO exposure during discrete 12 month follow-up periods with multivariable logistic regression.

Follow-up period (n)	Univariate		Multivariable	
	Odds Ratio (95% CI)	p value	Odds Ratio (95% CI)	p value
0 to 12 months (4489)	1.78 (1.49 to 2.11)	<0.001	1.63 (1.34 to 1.98)	<0.001
12 to 24 months (4489)	1.58 (1.24 to 2.01)	<0.001	1.42 (1.08 to 1/85)	0.011
24 to 36 months (3766)	1.64 (1.19 to 2.26)	0.003	1.53 (1.07 to 2.20)	0.02
36 to 48 months (2995)	2.37 (1.52 to 3.69)	<0.001	2.01 (1.023 to 3.30)	0.006
48 to 60 months (2253)	2.36 (1.36 to 4.09)	0.002	1.84 (1.00 to 3.39)	0.05
Multivariable model adjusted for age, sex, diagnosis, forensic status, antipsychotic route and year of study entry				

For peer review only

For peer review only - http://bmjopen.bmj.com/site/about/guidelines.xhtml

Community Treatment Orders: A controlled electronic case register study
Supplementary Material

Figure S1 Kaplan-Meier survival graph.

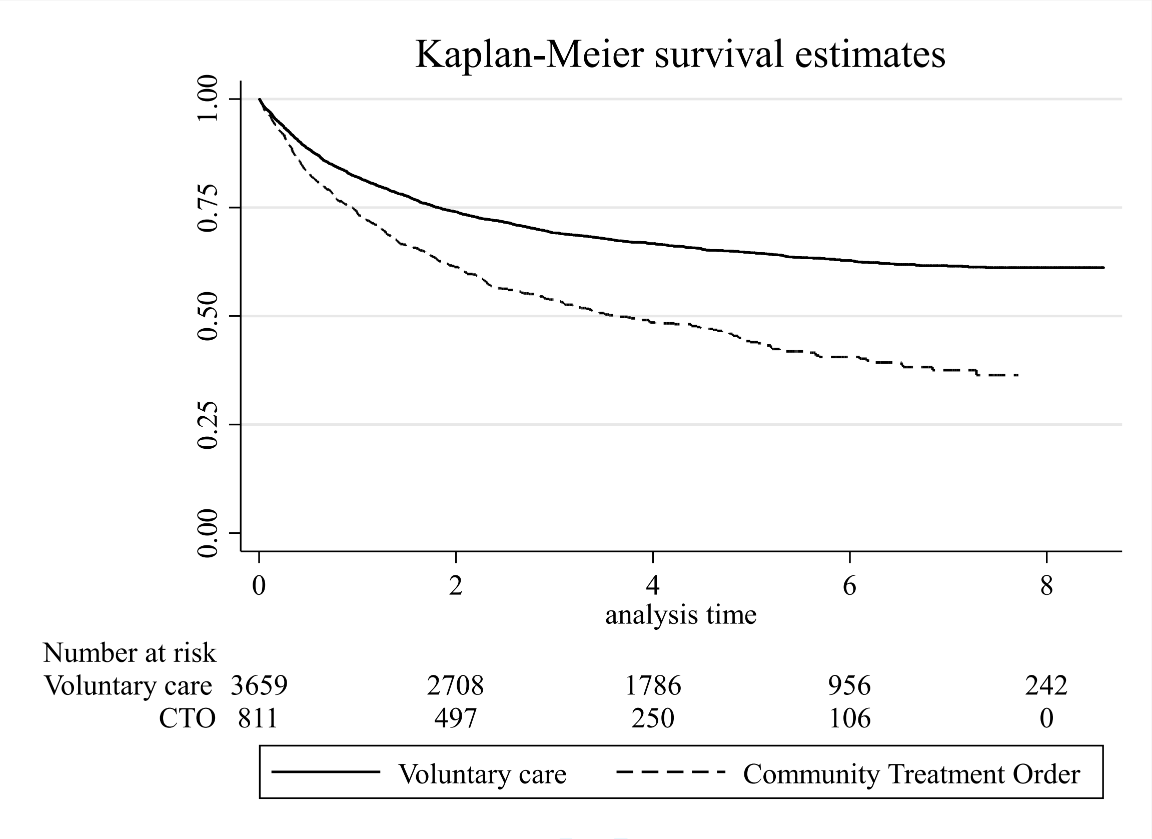

view only

For peer review only - http://bmjopen.bmj.com/site/about/guidelines.xhtml

Community Treatment Orders: A controlled electronic case register study
Supplementary Material

Figure S2 Proportion of individuals with either a reduction or an increase in the amount of days spent in inpatient care in the 24 months before and after index admission, by CTO exposure (n = 4460).

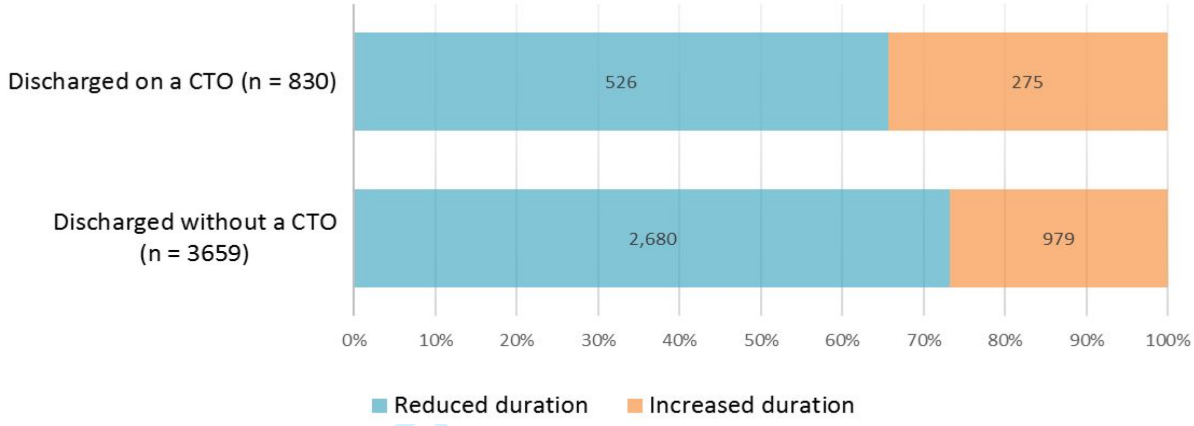

peer review only

BMJ Open

Community Treatment Orders and associations with readmission rates and duration of psychiatric hospital admission: A controlled electronic case register study

Journal:	*BMJ Open*
Manuscript ID	bmjopen-2019-035121.R1
Article Type:	Original research
Date Submitted by the Author:	26-Nov-2019

Complete List of Authors:	Barkhuizen, Wikus; King's College London, Department of Psychosis Studies<br>Cullen, Alexis; King's College London, Department of Psychosis Studies<br>Shetty, Hitesh; South London and Maudsley NHS Foundation Trust, Biomedical Research Centre Nucleus<br>Pritchard, Megan; Kings College London, Institute of Psychiatry, Psychology and Neuroscience<br>Stewart, Robert; King's College London, Institute of Psychiatry<br>McGuire, Philip; King's College London, Department of Psychosis Studies<br>Patel, Rashmi; King's College London, Department of Psychosis Studies

**Primary Subject Heading**:	Mental health
Secondary Subject Heading:	Health informatics, Epidemiology, Health services research
Keywords:	MENTAL HEALTH, PSYCHIATRY, EPIDEMIOLOGY, Health policy < HEALTH SERVICES ADMINISTRATION & MANAGEMENT, MEDICAL LAW, Health informatics < BIOTECHNOLOGY & BIOINFORMATICS

SCHOLARONE™ Manuscripts

Community Treatment Orders and associations with readmission rates and duration of psychiatric hospital admission: A controlled electronic case register study

Wikus Barkhuizen [1], Alexis E. Cullen [1], Hitesh Shetty [2], Megan Pritchard [1,2], Robert Stewart [1,2], Philip McGuire [1,2] and Rashmi Patel [1,2]

1. King's College London, Institute of Psychiatry, Psychology and Neuroscience, London, UK
2. South London and Maudsley NHS Foundation Trust, NIHR Maudsley Biomedical Research Centre, London, UK

Correspondence to:

Rashmi Patel

Tel: +44 (0) 2078480355

E-mail: bmj@rpatel.co.uk

For peer review only

Word count:

Abstract: 298

Total: 4,375

For peer review only

Competing interests: All authors have completed the ICMJE uniform disclosure form at www.icmje.org/coi_disclosure.pdf and declare: RS has received research funding from Roche, Janssen, GSK and Takeda. The other authors declare no competing interests.

Contributors: The study was conceived by AEC and RP. Data extraction was performed by WB with support from HS and MP. Statistical analyses and reporting of findings were carried out by WB, supervised by AEC and RP. WB, AEC, HS, MP, RS, PM and RP contributed to study design, manuscript preparation and approved the final version.

Ethics approval: The CRIS data resource received ethical approval as an anonymised dataset for secondary analyses from Oxfordshire REC C (Ref: 08/H0606/71+5).

Source of funding: HS, MP, RS and PM receive funding from the National Institute for Health Research (NIHR) Biomedical Research Centre at South London and Maudsley NHS Foundation Trust and King's College London, which also supports the development and maintenance of the BRC Case Register. RP has received support from a Medical Research Council (MRC) Health Data Research UK Fellowship (MR/S003118/1) and a Starter Grant for Clinical Lecturers (SGL015/1020) supported by the Academy of Medical Sciences, The Wellcome Trust, MRC, British Heart Foundation, Arthritis Research UK, the Royal College of Physicians and Diabetes UK. AEC has received support from a Sir Henry Wellcome Postdoctoral Fellowship (107395/Z/15/Z).

Role of funder: The views expressed are those of the authors and not necessarily those of the NHS, the NIHR or the Department of Health. The funders had no role in the design and conduct of the study; collection, management, analysis, and interpretation of the data;

For peer review only

preparation, review, or approval of the manuscript; and decision to submit the manuscript for publication.

Data sharing: The data accessed by CRIS remain within an NHS firewall and governance is provided by a patient-led oversight committee. Subject to these conditions, data access is encouraged and those interested should contact RS (robert.stewart@kcl.ac.uk), CRIS academic lead.

For peer review only

Abstract

Objectives: Limited evidence is available regarding the effect of Community Treatment Orders (CTOs) on mortality and readmission to psychiatric hospital. We compared clinical outcomes between patients placed on CTOs to a control group of patients discharged to voluntary community mental healthcare.

Design and setting: An observational study using de-identified electronic health record data from inpatients receiving mental healthcare in South London using the Clinical Record Interactive Search (CRIS) system. Data from patients discharged between November 2008 and May 2014 from compulsory inpatient treatment under the Mental Health Act were analysed.

Participants: 830 participants discharged on a CTO (mean age 40 years; 63% male) and 3,659 control participants discharged without a CTO (mean age 42 years; 53% male).

Outcome measures: The number of days spent in the community until readmission, the number of days spent in inpatient care in the two years prior to and the two years following the index admission, and mortality.

For peer review only

Results: The mean duration of a CTO was 3.2 years. Patients receiving care from forensic psychiatry services were 5 times more likely and patients receiving a long-acting injectable antipsychotic were twice as likely to be placed on a CTO. There was a significant association between CTO receipt and readmission in adjusted models (HR: 1.60, 95% CI 1.42 to 1.80, p<0.001). Compared to controls, patients on a CTO spent 17.3 additional days (95% CI 4.0 to 30.6, p=0.011) in a psychiatric hospital in the 2 years following index admission and had a lower mortality rate (HR: 0.66, 95% CI 0.50 to 0.88, p=0.004).

Conclusions: Many patients spent longer on CTOs than initially anticipated by policy makers. Those on CTOs are readmitted sooner, spend more time in hospital and have a lower mortality rate. These findings merit consideration in future amendments to the UK Mental Health Act.

Strengths and limitations of this study

For peer review only

This large observational study of CTOs is the first in England and Wales to include an appropriate control group to allow for robust evaluation of outcomes.

The use of clinical records allowed for evaluation of CTOs in real-world clinical practice, an advantage when it comes to generalisability of the findings.

The design of this study limited the risk of follow-up bias since all patients within the catchment area could be included in the analyses.

While we were able to account for several factors associated with the receipt of CTOs, we were not able to account for differences in illness severity between CTO and control patients.

For peer review only

Introduction

Community Treatment Orders (CTOs) were introduced in England and Wales under the 2007 Mental Health Act (MHA) to allow for compulsory clinical monitoring of people with serious mental disorders within community care settings and to facilitate recall to a psychiatric hospital following suspected relapse. Patients on a CTO who are recalled to hospital must be reassessed within 72-hours and where longer inpatient treatment is deemed necessary, the CTO may be revoked and the patient may remain admitted as an inpatient under the MHA section through which they were hospitalised prior to being discharged on a CTO. Only patients already hospitalised involuntarily for treatment of a mental disorder are eligible for a CTO. Orders require renewal every 6 months and, if not extended, lapse at which point the patient is effectively discharged from compulsory treatment. Despite initial scepticism from mental health practitioners,[1] their use has exceeded initial government projections[2] with around 5,000 currently being issued per year.[3]

Evidence from England and Wales on the effectiveness of CTOs is limited: The only randomised controlled trial (RCT) found no evidence that CTOs reduce

For peer review only

readmission rates[4 5] Two US trials reached contrary conclusions on the efficacy of CTOs reducing readmission rates.[6 7] Like the RCT conducted in England and Wales, these trials were unable to include forensic patients, thereby limiting generalisability. A Cochrane review of these RCTs found no support that CTOs reduce the rates of hospital readmissions.[8] Most observational studies conducted in England and Wales[9-11] found support for their use in reducing readmission rates and days spent in hospital. However, these studies were small and underpowered, examined outcomes within the first year only, and failed to include a control group. As such, the effects of CTOs on readmission rates and duration of readmission episodes are likely to have been overestimated. Most reviews of international studies conclude that the evidence on the effectiveness of CTOs is mixed and generally do not support improvements in readmission rates or duration of impatient treatment.[1 12-15] A recent meta-analysis of CTO studies concluded that studies without a control condition found evidence for a reduction in readmission rates and bed-days whilst those with a control condition did not.[16] Evidence from controlled studies on CTOs in England and Wales is currently lacking. Due to differences between jurisdictions in health care systems, compulsory community treatment programmes and legislation, findings

For peer review only

from previous controlled studies in other countries may not be generalisable to England and Wales.

Aims

We sought to gather data from a large provider of secondary mental healthcare in South London using electronic health record data for all patients who were involuntarily admitted to a psychiatric hospital since the introduction of CTOs in 2008. We aimed to estimate of the association of CTOs with hospital readmission by including an appropriate control group of those discharged to voluntary community care whilst adjusting for the demographic and clinical features associated with CTO receipt. Second, we aimed to compare the number of days spent in inpatient care in the two years following the index discharge between patients discharged on CTOs compared to those discharged voluntarily.

Methods

Study population and data collection

For peer review only

Data were obtained from de-identified electronic health records of mental health services provided by the South London and Maudsley National Health Service (NHS) Foundation Trust (SLaM). Serving a catchment of 1.3 million residents in the boroughs of Lambeth, Southwark, Lewisham and Croydon, SLaM provides more than 230 services that include specialist psychosis services, inpatient wards, community and outpatient services. SLaM treats an estimated 5,300 inpatients and 45,000 outpatients per year. The Clinical Record Interactive Search (CRIS) system[17 18] collates de-identified patient data from SLaM and contains both structured and free text fields of routine clinical data including case notes and correspondence.[19]

The analysed sample consisted of all SLaM patients who received and completed an involuntary inpatient episode under Section 3 or Section 37 of the MHA between November 2008 and May 2014, allowing for a follow-up period in all cases by using data up to July 2016.

The authors assert that all procedures contributing to this work comply with the ethical standards of the relevant national and institutional committees on human experimentation and with the Helsinki Declaration of 1975, as revised in 2008. All

For peer review only

procedures involving human subjects/patients were approved by the CRIS Oversight Committee which is responsible for ensuring all secondary research applications comply with legal requirements and ethical approval obtained from the Oxfordshire Research Ethics Committee C (reference 18/SC/0372).

Patient and public involvement

No patient involved.

Measures

Exposure to a community treatment order

The exposure was defined as discharge from an involuntary psychiatric hospital admission under Section 3 or Section 37 of the MHA to community care under a CTO and was compared to a control group of patients discharged to voluntary community care. CTO status and psychiatric hospital admission data were gathered from structured fields within the CRIS dataset. A manual free-text search on 50 random cases was conducted to confirm that no CTO orders were missed using data from these structured fields. 471 (12.9%) control patients and 140 (16.9%) CTO patients were discharged from more than one involuntary psychiatric hospital

For peer review only

admission during the study period. 359 patients in the CTO group (43.3%) had been placed on a CTO more than once. For patients who had never been placed on a CTO but had more than one involuntary admission under Section 3 or Section 37 of the MHA, the most recent Section 3 or 37 discharge date was taken as the index date. Where a patient had more than one CTO the most recent discharge to CTO was taken as the index date.

Covariates

Demographic and clinical information was gathered to identify factors associated with CTO receipt. Sex, ethnicity, age at the time of the index admission, the mode of involuntary admission under which patients were admitted (either Section 3 issued by an approved mental health practitioner (AMHP) and two doctors or Section 37 issued by criminal courts) and forensic status (determined if individuals were discharged from a forensic ward or to a forensic service) were collected from structured fields. Collected data included the start and end date of the index admission and the duration of the index admission in days. Psychiatric diagnosis was obtained from both structured fields on CRIS and free text searches to improve

For peer review only

the reliability of information at the time of the index episode. Diagnoses were subsequently grouped according to ICD-10 codes.[20]

We extracted the three most recent antipsychotic medications prescribed prior to discharge and method of administration from structured fields. A variable indexing antipsychotic type (oral, long-acting injectable [LAI], or none) was created. Those on more than one antipsychotic were classified in the LAI group if any of the most recently prescribed drugs two weeks prior to discharge were administered as LAI (to take into account combined oral and LAI treatment during initiation of LAI). We chose to distinguish between oral and LAI antipsychotics as LAI antipsychotics are associated with different rates of psychiatric hospital readmission compared to oral antipsychotics.

We included the year of study entry as a covariate as a measure of changes in mental healthcare service provision over time which may have been independently associated with risk of psychiatric hospital admission and number of inpatient days.

Outcomes of Community Treatment Orders

For peer review only

The primary outcome measure was the number of days spent in the community from the discharge date of the index hospital admission until readmission to a psychiatric ward or the end of the follow-up period (31st July 2016). We extracted data on mortality and censored individuals who died during the follow-up period in survival analyses. We additionally measured the number of days spent in inpatient care in the two years prior to and the two years following the index admission.

Missing Data

There were no missing data for predictor (CTO status) or outcome (readmission/number of inpatient days) variables. The number of patients with missing data for each covariate entered into multivariable analyses was as follows: age: 41; sex: 0; diagnosis: 43; forensic status: 198; antipsychotic route: 107; year of study entry: 0; number of inpatient days in 2 years prior to index admission: 0. Where patients had missing covariate data, they were dropped from multivariable analyses. 92.3% of patients had complete covariate data.

Analyses

For peer review only

All analyses were conducted in Stata 13[21] and statistical significance was defined at $p<0.05$. Univariate logistic regression models were first performed to assess the association between CTO status and demographic/clinical factors (sex, age, ethnicity, diagnosis, forensic status, the mode of admission and the route of administration of antipsychotic medication). A fully adjusted model using multivariable logistic regression was subsequently performed with all covariates entered simultaneously to predict CTO status.

To adjust for censoring based on the study follow-up period, mean and median CTO duration were estimated using the “stci” and “stsum” commands in STATA censored on 31st July 2016 (the end of the study follow-up window).

Kaplan-Meier curves and log-rank tests were used to assess differences in the observed survival rates (time to next admission) between CTO and voluntarily discharged patients. To assess the association between CTO exposure and time to next admission, unadjusted Cox regression models were performed. Covariates that were significantly associated with CTO exposure in the adjusted logistic regression analyses were then added to the Cox regression in a multivariable model on the

For peer review only

basis that factors significantly associated with CTO exposure may be, *a priori*, most influential on associations with clinical outcomes. Overall significance of the Cox regression model was assessed using the −2 log-likelihood ratio test. A Schoenfeld residuals test and visual inspection of Nelson-Aalen cumulative hazard curves were used to test the proportional-hazards assumption. An additional discrete time analysis was performed where the proportional-hazards assumption was not met.

The CTO and control group were compared on the number of days spent in a psychiatric hospital two years after the index date using multiple linear regression with the same covariates as used in the multivariable Cox regression on time to readmission plus an additional covariate measuring the number of days spent in inpatient care two years prior to the index admission as this factor was associated with increased number of days spent in a psychiatric hospital subsequently (Pearson coefficient: 0.23, $p<0.001$).

Results

For peer review only

The cohort comprised 4,489 SLaM patients discharged from involuntary inpatient care between 2008 and 2014. Of these, 830 (18.5%) were placed on a CTO at least once while the remaining 3659 (81.5%) were not. The mean age at discharge of those on CTOs was 39.5 years (s.d. = 13.3) and those discharged without a CTO 42.1 years (s.d. = 16.4). The CTO group had a larger proportion of males compared to those not on a CTO (62.9% v 52.8%). Table 1 summarises the demographic details of the sample by CTO exposure.

The mean duration of CTO was 3.20 years (95% CI 2.95 to 3.45). The median duration of CTO was 2.65 years (IQR 0.50 to 5.65). Of those whose CTOs ended before the end of the study period (n = 434), 19.0% were discharged to standard care, 15.7% had their CTOs revoked and were readmitted to hospital under their previous MHA Section, and the CTOs of 12.4% of patients lapsed due to not being actively renewed at the required 6-monthly review.

489 patients died during the study period. Of these, 432 were in the control group and 57 were in the CTO group. The overall mortality rate was 2.14% per year. The mortality rate for the control group was 2.27% per year. The mortality rate for the

For peer review only - http://bmjopen.bmj.com/site/about/guidelines.xhtml

CTO group was 1.53% per year (hazard ratio compared to controls 0.66, 95% CI 0.50 to 0.88, p=0.004).

A breakdown of year of study entry is provided in the online supplementary table S1 and indicates that rates of CTO usage have varied over time with peak usage occurring in 2013.

Predictors of receipt of a CTO

In unadjusted logistic regression models (table 1), several significant predictors of CTO exposure were identified. Females had lower odds than males of being placed on a CTO. Those between 25-34 years of age more likely to receive a CTO, black patients had higher odds of receiving a CTO compared to white patients. Compared to receiving a diagnosis of schizophrenia, those with other diagnoses had reduced odds of being placed on a CTO. The odds of receiving a CTO were five times higher among those receiving care from forensic services compared to those in secondary (non-forensic) care and 2.3 times higher in those on LAI relative to those on oral antipsychotics. Patients on a Section 37 (typically issued by criminal courts) during

For peer review only

their index inpatient episode had twice the odds of being placed under a CTO compared to patients on a Section 3.

A logistic regression model that adjusted for all predictors (table 1; $\chi^2(15) = 320.58$, $p < 0.001$; pseudo-$R^2 = 0.08$) indicated that all factors reported above, with the exception of patient ethnicity, remained significantly associated with CTOs, albeit with attenuated odds ratios.

Time to readmission

The overall mean time to re-admission was 5.55 years (95% CI 5.44 to 5.66). The mean time to re-admission for the control group was 5.82 years (95% CI 5.70 to 5.94). The mean time to re-admission for the CTO group was 4.02 years (95% CI 3.80 to 5.25). Kaplan-Meier curves (see online supplementary figure S1) comparing the observed survival time to next admission indicated that patients on a CTO were likely to be readmitted sooner than patients who were not on a CTO (univariate hazard ratio: 1.76, 95% CI 1.58 to 1.96, p<0.001). A multivariable Cox regression model (table 2) confirmed the association between CTO exposure and reduced time to readmission which remained significant after adjusting for covariates. Figure 1

For peer review only

illustrates the survival rates depending on CTO status. The Schoenfeld residuals test indicated that the proportional hazards assumption was not met (χ^2 =14.3, p<0.001) suggesting that the effect of CTO exposure on time next admission was not constant over time. We therefore conducted a discrete time analysis using multivariable logistic regression on discrete periods of 12 months of follow-up (online supplementary table S2) that confirmed the association between CTO receipt and reduced time to next readmission across different periods of follow-up.

Length of stay

Patients not on a CTO spent a mean of 148.3 days (std dev: 164.1) and median of 85 days (IQR 43 to 183) in a psychiatric hospital in the two years after the index date. Patients who were on a CTO spent a mean of 177.7 days (std dev: 178.4) and median of 116.5 days (IQR 52 to 253) in a psychiatric hospital in the two years after the index date. After adjusting for covariates in multiple linear regression (table 3), Those on CTOs spent 17.3 additional days (95% CI 4.0 to 30.6) in a psychiatric hospital compared to patients not on a CTO.

For peer review only - http://bmjopen.bmj.com/site/about/guidelines.xhtml

Online supplementary figure S2 illustrates that a greater proportion of patients who were not placed on a CTO spent less time in hospital compared to prior to their index admission than patients who were placed on a CTO.

For peer review only

Discussion

Main findings

To the best of our knowledge, this is the largest observational study to evaluate the predictors and outcomes of CTOs in England and Wales. Importantly, the inclusion of a control group of patients discharged to voluntary care allowed for a robust evaluation of CTOs in real clinical settings. Those placed on CTOs were more likely to be male, in the 25-34 age group, have a diagnosis of schizophrenia-spectrum or depressive disorders, have been treated in forensic mental health services, and in receipt of LAI antipsychotics. The greatest proportion of admissions resulting in discharge to a CTO occurred in 2013. CTOs were associated with a higher rate of readmission, even after accounting for associated demographic and clinical features. Patients on a CTO had a greater number of days spent in psychiatric hospital in the 2 years following discharge from index admission than patients not on a CTO.

Community Treatment Orders and relapse rates

For peer review only

We found that, at any given time during the follow-up period, those on CTOs had an increased risk of being readmitted to psychiatric inpatient treatment compared to those voluntarily discharged after controlling for factors associated with CTO status. Previous studies in England and Wales[9-11] reported a reduction in hospital days and admission rates after patients were placed on a CTO compared to the two years prior to the order. These studies employed uncontrolled before-and-after designs on samples ranging from 20 to 37 patients, all recruited from a single service. Consequently, both statistical power and the generalisability of findings were limited. Also impacting on the generalisability, one study[9] excluded those on short duration CTOs. In the absence of control conditions, effects other than CTO exposure, including the natural trajectory of disease and other treatment effects, are more likely to have accounted for some of the improvement in the rates and duration of readmissions previously reported.

There are several possible explanations for why those on CTOs had higher rates of psychiatric hospital admission compared to controls in our study. One plausible explanation is that patients with more severe symptoms or a history of relapse were more likely to receive CTOs. Hospitalisation rates may also be higher

For peer review only - http://bmjopen.bmj.com/site/about/guidelines.xhtml

due to the relative ease of readmission of deteriorating patients under the CTO pathway, which does not require a Mental Health Act assessment to be conducted. An alternative possibility is that CTOs were not effective at reducing relapse rates. Whilst we controlled for factors associated with receipt of CTOs (forensic status, diagnosis and the route of antipsychotic administration), we were not able to account for possible differences in illness severity between CTO patients and controls, nor did we compare relapse rates prior to and after being placed on CTOs.

Our study also examined CTO outcomes in terms of differences in the number of days spent in psychiatric hospitals. After adjusting for covariates including the amount of time spent in a psychiatric hospital prior to index admission, those on CTOs spent 17 additional days in a psychiatric hospital during the follow-up period compared to patients who were not placed on a CTO. Our findings are in line with previous studies that have examined the number of days spent in hospital pre- and post-CTO exposure.[9-11]; however, these studies failed to include a control group.

The duration of Community Treatment Orders

We report that, once a CTO is issued, many patients are kept under this treatment option for extensive periods. The mean duration of a CTO in our study was 3 years, far exceeding initial government projections of 9 months.[22] This is informative because while official figures on the number of CTOs issued per year are readily available,[3] national statistics on their duration are hard to come by. In the current sample, CTOs also lasted longer than previously found in samples from England and Wales. For instance, we found that CTOs lasted three times as long compared to Dye et al[11] who reported an average duration of 52.6 weeks (s.d. = 31.7). A likely reason for this may be the longer follow-up period in this study; At the end of Dye et al's 2-year follow-up period, 35% of their sample were still on CTOs. That a large proportion of the eligible patient population are exposed to compulsory community treatment, often for long periods of time, underscores the importance of understanding the characteristics of and outcomes for those on CTOs.

Demographic and clinical features of patients who receive Community Treatment Orders

The age and sex distributions within the CTO subsample were similar to previous reports.[10 11 23] Unlike previous studies, we were able to compare the demographics of those on CTOs to those discharged without a CTO: Taking into account other factors, those already hospitalised under the MHA were 4.4 times more likely to receive a CTO if they were in forensic services, twice as likely to be on LAI than oral antipsychotics, more likely male and with a diagnosis of a schizophrenia-spectrum disorder compared to bipolar, other psychotic or miscellaneous disorders (but not depression).

Almost 20% of those placed on CTOs were either hospitalized within forensic wards or discharged to forensic services. Consequently, those with forensic service involvement were more likely than non-forensic patients to be subject to compulsory treatment. Considering that the most commonly cited reasons why patients are placed on CTOs in England and Wales relates to clinical considerations such as improving adherence to treatment,[24] as opposed to monitoring risk, this result is somewhat surprising. It is possible that CTOs may have been used primarily for protective or monitoring reasons among forensic patients more commonly than previously suggested. In the absence of further investigation into the motivations for

For peer review only

issuing CTOs and their conditions within this sample, it is not clear what underlies the reason that forensic status was found to be predictive of being placed on a CTO.

The proportion of patients of black ethnicity being placed on CTOs was similarly high to that reported by Patel et al[23] in their cohort within the same geographic area of London. Patel et al. speculated that their finding may have been due to the high number of black patients sectioned under the MHA. The current study was able to explore this phenomenon in further detail as it included all those detained under the MHA and found that black patients who were already in psychiatric hospitals were significantly more likely to be placed on a CTO than other patients. However, the association between ethnicity and CTO exposure did not remain significant after considering other predictors of being started on a CTO, including forensic, clinical and demographic characteristics, suggesting that these factors explained the positive association we observed between black ethnicity and CTO receipt.

Like others, this study found that a large proportion of those on CTOs were prescribed LAI formulation antipsychotics (41%).[9 10 23 25] Moreover, this study

For peer review only - http://bmjopen.bmj.com/site/about/guidelines.xhtml

demonstrated that those on a CTO had twice the odds of being prescribed LAIs than those not on a CTO, a finding that remained significant after controlling for the effect of diagnosis. Several reasons for a preference by psychiatrists for prescribing LAI formulation antipsychotics to those on CTOs have been proposed: Patel et al[23] suggested that those less likely to adhere are placed on LAI and therefore at greater risk of relapse. This would make them likely candidates for a CTO. Adherence to LAIs is also easier to monitor and therefore preferable for reasons of enforcement.

The significantly lower mortality rate among those who received CTOs during our study period is in line with similar reports that compared mortality rates between CTO and control patients in Australia.[26 27] Kisely et al[26] suggested that the differences in mortality rates in their study may be due to closer follow-up in the community among patients on CTOs. This may enable better detection and treatment of emerging physical health disorders and highlights the importance of robust community follow-up for patients following discharge from psychiatric hospital regardless of CTO status.

For peer review only

Strengths and limitations

The advantage of using health records to investigate the use and effects of CTOs is that they provide data which represents real-world clinical practice which may be more generalisable than findings from RCTs whose participants may not be representative of the wider population receiving mental healthcare. Additionally, follow-up bias is limited as most subjects are included at all time points of data gathering unless they leave the area or die.[28 29] The SLaM BRC CRIS system has specific advantages over many other electronic case registers: with access to over 400,000 patient records and combining structured fields and free-text searches from fully digitised case registers, it has both the quality of smaller databases and the quantity of data only large registers provide.[17] On the other hand, the use of routine clinical data limits the possible number of covariates that can be reliably controlled for and does not rule out confounding by indication. For instance, this study did not control for illness severity, urbanicity, migration status, educational attainment or socioeconomic characteristics. Electronic patient records are also susceptible to erroneous or partial recording of treatment events. During the study period patients may have moved out of the catchment area or into the catchment area from

For peer review only

elsewhere. As the data source only included records from SLaM, CTO and hospital admission episodes in other mental healthcare providers would not have been included.

Using the most recent CTO episode that patients were subject to (in cases where the same patient was subject to more than one CTO during the window period) enabled the study to report on current practices; the way in which CTOs have been used in other jurisdictions has indicated possible change over time as practitioners become more familiar with the legislation.[30]

The rates of readmission reported here should be interpreted with some cautions as our survival analysis did not meet the assumption of proportional hazards. However, discrete time analysis indicated that the association of CTOs with increased rates of readmission persisted across all periods of follow-up.

Implications

There are several implications for both current and future research into the effects of CTOs. We found that patients spend several years on a CTO, far longer

For peer review only

than the original projected duration of 9 months. At the same time, our study demonstrated that patients on CTOs have greater rates of readmission and spend longer in psychiatric hospital than patients who are not on CTOs, going against previously published studies which did not include a control group.

We found that certain demographic and clinical features, such as ethnicity and forensic background, are strongly associated with CTO exposure. Several observations on the use of CTOs warrant further investigation, including the influence of LAIs on outcomes, and the effects of extensive periods of community compulsion on patient engagement.

We found a higher incidence of CTOs among black patients adding to previous findings which indicate that black and minority ethnic (BME) groups are overrepresented in compulsory mental healthcare admission and treatment.

In conclusion, our study found that patients may spend several years on a CTO and that CTOs are not associated with a reduction in psychiatric hospital admission or with less time spent in a psychiatric hospital. The implementation of

For peer review only

CTOs in future amendments of the UK Mental Health Act should be reviewed in light of these findings.[31]

For peer review only

Author details

Wikus Barkhuizen (MSc), Postgraduate student, King's College London (Institute of Psychiatry, Psychology and Neuroscience), London, UK

Alexis Cullen (PhD), Sir Henry Wellcome Postdoctoral Fellow, King's College London (Institute of Psychiatry, Psychology and Neuroscience), London, UK

Hitesh Shetty (MSc), Clinical Informatician, Maudsley Biomedical Research Centre, South London and Maudsley NHS Foundation Trust, London, UK

Megan Pritchard (MSc), Training and Development Lead, Maudsley Biomedical Research Centre, South London and Maudsley NHS Foundation Trust, London, UK

Robert Stewart (MD), Professor of Psychiatric Epidemiology & Clinical Informatics, King's College London (Institute of Psychiatry, Psychology and Neuroscience), London, UK

Philip McGuire (MD PhD), Professor of Psychiatry & Cognitive Neuroscience, King's College London (Institute of Psychiatry, Psychology and Neuroscience), London, UK

For peer review only

Rashmi Patel (BM BCh PhD), MRC Health Data Research UK Fellow, King's College London (Institute of Psychiatry, Psychology and Neuroscience), London, UK

For peer review only

References

1. Churchill R. International experiences of using community treatment orders (PhD diss.). Kings College London, 2007.

2. Trevithick L, Carlile J, Nodiyal S, *et al*. Community treatment orders: an analysis of the first five years of use in England. *Br J Psychiatry* 2018;**212**:175-79.doi: 10.1192/bjp.2017.51

3. NHS Digital. Mental Health Act Statistics, Annual Figures: 2016-17, Experimental statistics, 2017. https://www.gov.uk/government/publications/independent-review-of-the-mental-health-act-interim-report (Accessed 8 Jul 2019).

4. Burns T, Rugkasa J, Molodynski A, *et al*. Community treatment orders for patients with psychosis (OCTET): a randomised controlled trial. *Lancet* 2013;**381**:1627-33.doi: 10.1016/s0140-6736(13)60107-5

5. Burns T, Yeeles K, Koshiaris C, *et al*. Effect of increased compulsion on readmission to hospital or disengagement from community services for patients with psychosis:

follow-up of a cohort from the OCTET trial. *Lancet Psychiatry* 2015;**2**:881-90. doi: 10.1016/s2215-0366(15)00231-x

6. Swartz MS, Swanson JW, Wagner HR, *et al*. Can involuntary outpatient commitment reduce hospital recidivism?: Findings from a randomized trial with severely mentally ill individuals. *Am J Psychiatry* 1999;**156**:1968-75.doi: 10.1176/ajp.156.12.1968
7. Steadman HJ, Gounis K, Dennis D, *et al*. Assessing the New York City involuntary outpatient commitment pilot program. *Psychiatr Serv* 2001;**52**:330-6.doi: 10.1176/appi.ps.52.3.330
8. Kisely SR, Campbell LA, O'Reilly R. Compulsory community and involuntary outpatient treatment for people with severe mental disorders. *Cochrane Database Syst Rev* 2017;**3**:Cd004408.doi: 10.1002/14651858.CD004408.pub5
9. Rawala M, Gupta S. Use of community treatment orders in an inner-London assertive outreach service. *Psychiatr Bull* 2014;**38**:13-8.doi: 10.1192/pb.bp.112.042184
10. Awara MA, Jaffar K, Roberts P. Effectiveness of the Community Treatment Order in streamlining psychiatric services. *J Ment Health* 2013;**22**:191-7.doi: 10.3109/09638237.2013.775408
11. Dye S, Dannaram S, Loynes B, *et al*. Supervised community treatment: 2-year follow-up study in Suffolk. *Psychiatrist Online* 2012;**36**:298-302.doi: 10.1192/pb.bp.111.036657
12. Rugkasa J. Effectiveness of Community Treatment Orders: The International Evidence. *Can J Psychiatry* 2016;**61**:15-24.doi: 10.1177/0706743715620415
13. Maughan D, Molodynski A, Rugkasa J, *et al*. A systematic review of the effect of community treatment orders on service use. *Soc Psychiatry Psychiatr Epidemiol* 2014;**49**:651-63.doi: 10.1007/s00127-013-0781-0

For peer review only

14. Kisely S, Campbell LA, Scott A, *et al*. Randomized and non-randomized evidence for the effect of compulsory community and involuntary out-patient treatment on health service use: systematic review and meta-analysis. *Psychol Med* 2007;**37**:3-14.doi: 10.1017/s0033291706008592

15. Kisely S, Hall K. An updated meta-analysis of randomized controlled evidence for the effectiveness of community treatment orders. *Can J Psychiatry* 2014;**59**:561-4.doi: 10.1177/070674371405901010

16. Barnett P, Matthews H, Lloyd-Evans B, *et al*. Compulsory community treatment to reduce readmission to hospital and increase engagement with community care in people with mental illness: a systematic review and meta-analysis. *Lancet Psychiatry* 2018;**5**:1013-22.doi: 10.1016/s2215-0366(18)30382-1

17. Perera G, Broadbent M, Callard F, *et al*. Cohort profile of the South London and Maudsley NHS Foundation Trust Biomedical Research Centre (SLaM BRC) Case Register: current status and recent enhancement of an Electronic Mental Health Record-derived data resource. *BMJ Open* 2016;**6**:e008721.doi: 10.1136/bmjopen-2015-008721

18. Stewart R, Soremekun M, Perera G, *et al*. The South London and Maudsley NHS Foundation Trust Biomedical Research Centre (SLAM BRC) case register: development and descriptive data. *BMC Psychiatry* 2009;**9**:51.doi: 10.1186/1471-244x-9-51

19. Fernandes AC, Cloete D, Broadbent MT, *et al*. Development and evaluation of a de-identification procedure for a case register sourced from mental health electronic records. *BMC Med Inform Decis Mak* 2013;**13**:71.doi: 10.1186/1472-6947-13-71

20. World Health Organization. The ICD-10 classification of mental and behavioural disorders: diagnostic criteria for research. Geneva: World Health Organization 1993.

For peer review only

21. StataCorp. Stata Statistical Software: Release 13. College Station, TX: StataCorp LP. 2013.

22. Department of Health. Mental Health Bill: Regulatory Impact Assessment, 2006. https://webarchive.nationalarchives.gov.uk/20081212000105/http://www.dh.gov.uk/en/Publicationsandstatistics/Legislation/Regulatoryimpactassessment/DH_076477 (Accessed 22 Dec 2018).

23. Patel MX, Matonhodze J, Baig MK, *et al*. Naturalistic outcomes of community treatment orders: antipsychotic long-acting injections versus oral medication. *J Psychopharmacol* 2013;**27**:629-37.doi: 10.1177/0269881113486717

24. DeRidder R, Molodynski A, Manning C, *et al*. Community treatment orders in the UK 5 years on: a repeat national survey of psychiatrists. *BJPsych Bull* 2016;**40**:119-23.doi: 10.1192/pb.bp.115.050773

25. Rugkasa J, Molodynski A, Yeeles K, *et al*. Community treatment orders: clinical and social outcomes, and a subgroup analysis from the OCTET RCT. *Acta Psychiatr Scand* 2015;**131**:321-9.doi: 10.1111/acps.12373

26. Kisely S, Preston N, Xiao J, *et al*. Reducing all-cause mortality among patients with psychiatric disorders: a population-based study. *CMAJ* 2013;**185**:E50-6.doi: 10.1503/cmaj.121077

27. Segal SP, Burgess PM. Effect of conditional release from hospitalization on mortality risk. *Psychiatr Serv* 2006;**57**:1607-13.doi: 10.1176/ps.2006.57.11.1607

28. Kisely S, Campbell LA, Preston NJ, *et al*. Can epidemiological studies assist in the evaluation of community treatment orders? - The experience of Western Australia and Nova Scotia. *Int J Law Psychiatry* 2006;**29**:507-15. doi: 10.1016/j.ijlp.2006.01.006

29. Swanson JW, Swartz MS. Why the evidence for outpatient commitment is good enough. *Psychiatr Serv* 2014;**65**:808-11.doi: 10.1176/appi.ps.201300424

For peer review only

30. Kisely S, Preston N, Xiao J, *et al*. An eleven-year evaluation of the effect of community treatment orders on changes in mental health service use. *J Psychiatr Res* 2013;**47**:650-6.doi: 10.1016/j.jpsychires.2013.01.010

31. Department of Health and Social Care. Independent review of the Mental Health Act: interim report, 2018. https://www.gov.uk/government/publications/independent-review-of-the-mental-health-act-interim-report (Accessed 19 Nov 2018).

For peer review only

Tables and Figure legends

Table 1 Sample characteristics and logistic regression of factors associated with Community Treatment Orders

	Total group (*N* = 4489)		Controls (*n* = 3659)		CTO (*n* = 830)		Unadjusted models			Adjusted models		
	n	%	*n*	%	*n*	%	OR	*CI*	*p*	OR	*CI*	*p*
**Sex**												
Male	2,453	54.6	1,931	52.8	522	62.9	*Ref*	-	-	-	-	-
Female	2,036	45.4	1,728	47.2	308	37.1	0.66	*0.56-0.77*	<.001	0.83	*0.71-0.99*	0.042
**Age group**												
<25	683	15.4	577	15.8	106	13.4	0.69	*0.54-0.89*	0.005	0.76	*0.57-1.01*	0.063
25-34	1045	23.5	826	22.6	219	27.8	*Ref*	-	-	-	-	-
35-44	1,027	23.1	825	22.6	202	25.6	0.92	*0.75-1.14*	0.466	0.91	*0.72-1.15*	0.415
45-55	880	19.8	700	19.1	180	22.8	0.97	*0.78-1.21*	0.786	0.94	*0.74-1.20*	0.642
> 55	813	18.3	731	20.0	82	10.4	0.42	*0.32-0.56*	<.001	0.48	*0.36-0.65*	<.001
**Ethnicity**												
White	1,728	38.5	1,449	39.6	279	33.6	*Ref*	-	-	-	-	-
Black	2,192	48.9	1,727	47.2	465	56.0	1.40	*1.19-1.65*	<.001	1.08	*0.89-1.30*	0.457
Other	566	12.6	480	13.1	86	10.4	0.93	*0.72-1.21*	0.591	0.79	*0.59-1.07*	0.134
**Primary diagnosis**												
Schizophrenia	2092	47.1	1,609	44.0	483	61.2	*Ref*	-	-	-	-	-
Other psychotic	628	14.1	544	14.9	84	10.7	0.51	*0.40-0.66*	<.001	0.60	*0.46-0.79*	<.001
Bipolar and related	630	14.2	554	15.2	76	9.6	0.46	*0.35-0.59*	<.001	0.59	*0.44-0.78*	<.001
Depressive disorders	428	9.6	365	10.0	63	8.0	0.57	*0.43-0.77*	<.001	0.93	*0.68-1.26*	0.629
Other	668	15.0	585	16.0	83	10.5	0.47	*0.37-0.61*	<.001	0.63	*0.47-0.84*	0.002
**Forensic status**												
Secondary care	3983	92.8	3318	95.6	665	81.1	*Ref*	-	-	-	-	-
Forensic patient	308	7.2	153	4.4	155	18.9	5.05	*3.98-6.42*	<.001	4.41	*3.40-5.72*	<.001
**Route of antipsychotic**												
Oral	3162	72.2	2741	74.9	421	58.2	*Ref*	-	-	-	-	-
LAI	1,142	26.1	844	23.1	298	41.2	2.30	*1.94-2.72*	<.001	2.18	*1.83-2.61*	<.001
None	78	1.8	74	2.0	4	0.6	0.35	*0.13-0.97*	0.043	0.53	*0.18-1.50*	0.23
**Mode of admission**												
Section 3	4284	96.3	3546	96.9	738	93.5	*Ref*	-	-	-	-	-
Section 37	164	3.7	113	3.1	51	6.5	2.17	*1.54-3.05*	<.001	1.42	*0.93-2.16*	0.105

Note: CTO = Community Treatment Orders; LAI = Long-acting injectable; Adjusted models accounting for all predictor variables entered simultaneously into multiple logistic models.

For peer review only

Table 2 Multivariable Cox regression analysis of time to next admission following discharge from index admission (*n* = 4,144)

**Factor**	**Hazard Ratio (95% CI)**	**p value**
**Control group (reference)**		
Community Treatment Order (CTO) group	1.60 (1.42 to 1.80)	<0.001
**Age (reference: <25 years)**		
25-34 years	1.02 (0.87 to 1.18)	0.84
35-44 years	0.89 (0.76 to 1.04)	0.13
45-55 years	0.80 (0.68 to 0.94)	0.007
> 55 years	0.65 (0.54 to 0.78)	<0.001
**Sex (reference: Male)**		
Female	0.95 (0.86 to 1.04)	0.27
**Diagnosis (reference: Schizophrenia)**		
Other psychotic disorder	0.90 (0.78 to 1.05)	0.18
Bipolar Disorder	1.23 (1.07 to 1.41)	0.004
Depressive disorders	1.19 (1.01 to 1.41)	0.041
Other	0.81 (0.68 to 0.95)	0.011
**Forensic status (reference: Not forensic)**		
In forensic services	1.12 (0.94 to 1.34)	0.22
**Antipsychotic route (reference: Oral)**		
Long-acting injectable (LAI/depot)	1.11 (0.99 to 1.23)	0.063
None	0.47 (0.25 to 0.85)	0.013
**Year of study entry (reference: 2008)**		
2009	1.20 (0.98 to 1.46)	0.083
2010	1.29 (1.06 to 1.58)	0.013
2011	1.47 (1.21 to 1.79)	<0.001
2012	1.81 (1.49 to 2.19)	<0.001
2013	2.02 (1.66 to 2.45)	<0.001
2014	2.45 (1.96 to 3.08)	<0.001
Analysis adjusted for all variables reported in this table		

For peer review only

Table 3 Multiple linear regression investigating number of inpatient days in a psychiatric hospital during the 2 years following discharge from index admission (*n* = 4,190)

Factor	β Coefficient (95% CI)	p value
**Control group (reference)**		
Community Treatment Order (CTO) group	17.3 days (4.0 to 30.6)	0.011
**Age (reference: <25 years)**		
25-34 years	-6.5 days (-22.4 to 9.5)	0.43
35-44 years	-23.7 days (-39.7 to -7.7)	0.004
45-55 years	-12.6 days (-29.2 to 4.0)	0.14
> 55 years	13.9 days (-2.9 to 30.8)	0.11
**Sex (reference: Male)**		
Female	-17.4 days (-27.2 to -7.7)	<0.001
**Diagnosis (reference: Schizophrenia)**		
Other psychotic disorder	-21.1 days (-35.5 to -6.7)	0.004
Bipolar Disorder	-40.2 days (-54.6 to -25.8)	<0.001
Depressive disorders	-33.6 days (-50.5 to -16.7)	<0.001
Other	28.7 days (13.7 to 43.8)	<0.001
**Forensic status (reference: Not forensic)**		
In forensic services	33.4 days (14.1 to 52.7)	0.001
**Antipsychotic route (reference: Oral)**		
Long-acting injectable (LAI/depot)	-15.8 days (-26.8 to -4.8)	0.005
None	29.0 days (-9.0 to 67.0)	0.14
**Year of study entry (reference: 2008)**		
2009	-13.0 days (-31.4 to 5.4)	0.17
2010	-9.2 days (-27.6 to 9.2)	0.33
2011	-12.6 days (-30.7 to 5.4)	0.17
2012	-17.6 days (-35.1 to -0.1)	0.049
2013	-3.8 days (-21.2 to 13.6)	0.67
2014	-3.5 days (-24.9 to 18.0)	0.75
Number of inpatient days in 2 years prior to start of index admission	0.31 days (0.27 to 0.35)	<0.001
Analysis adjusted for all variables reported in this table		

Figure 1 Graph of cox survival function comparing time to next admission between CTO and controls (*n* = 4,439)

For peer review only

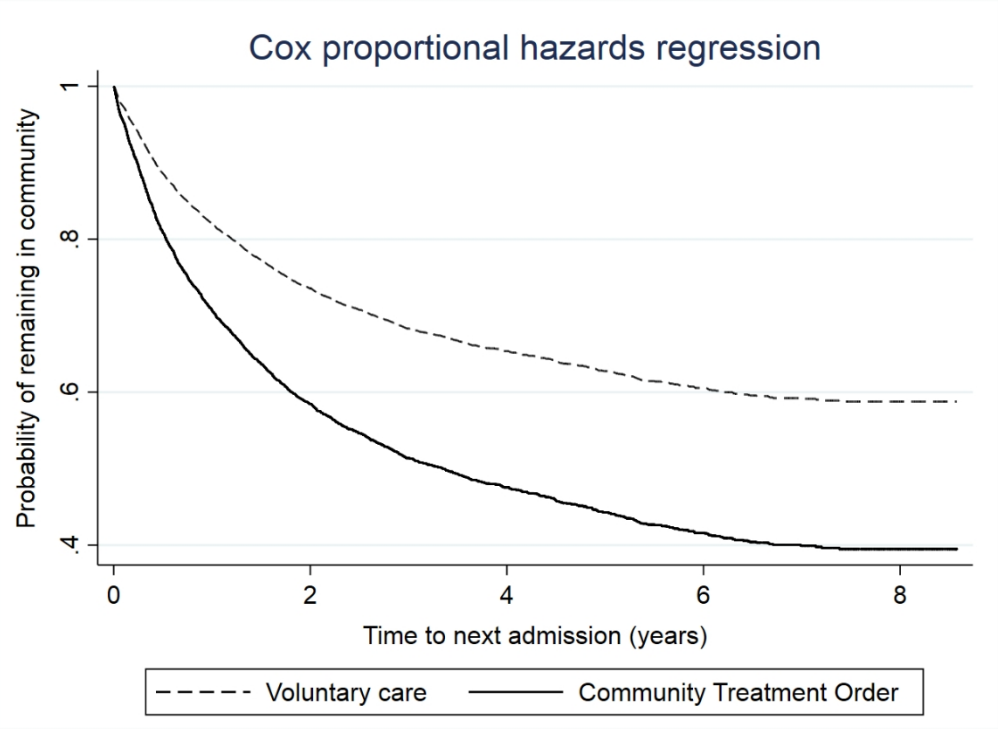

Graph of cox survival function comparing time to next admission between CTO and controls (n = 4,439)

Community Treatment Orders: A controlled electronic case register study
Supplementary Material

Table S1 Number of patients in CTO and control group by year of study entry (*n* = 4,489)

Year of study entry	Control group n (%)	CTO group n (%)	Total
2008	560 (96.2%)	22 (3.8%)	582
2009	522 (81.3%)	120 (18.7%)	642
2010	503 (79.2%)	132 (20.8%)	635
2011	556 (80.6%)	134 (19.4%)	690
2012	615 (80.6%)	148 (19.4%)	763
2013	625 (75.7%)	201 (24.3%)	826
2014	278 (79.2%)	73 (20.8%)	351
Total	3659 (81.5%)	830 (18.5%)	4489
χ^2 = 106.6, p<0.001			

Table S2 Discrete time analysis predicting time to next admission by CTO exposure during discrete 12 month follow-up periods with multivariable logistic regression.

Follow-up period (n)	Univariate		Multivariable	
	Odds Ratio (95% CI)	p value	Odds Ratio (95% CI)	p value
0 to 12 months (4489)	1.78 (1.49 to 2.11)	<0.001	1.63 (1.34 to 1.98)	<0.001
12 to 24 months (4489)	1.58 (1.24 to 2.01)	<0.001	1.42 (1.08 to 1/85)	0.011
24 to 36 months (3766)	1.64 (1.19 to 2.26)	0.003	1.53 (1.07 to 2.20)	0.02
36 to 48 months (2995)	2.37 (1.52 to 3.69)	<0.001	2.01 (1.023 to 3.30)	0.006
48 to 60 months (2253)	2.36 (1.36 to 4.09)	0.002	1.84 (1.00 to 3.39)	0.05
Multivariable model adjusted for age, sex, diagnosis, forensic status, antipsychotic route and year of study entry				

For peer review only

For peer review only - http://bmjopen.bmj.com/site/about/guidelines.xhtml

Community Treatment Orders: A controlled electronic case register study
Supplementary Material

Figure S1 Kaplan-Meier survival graph.

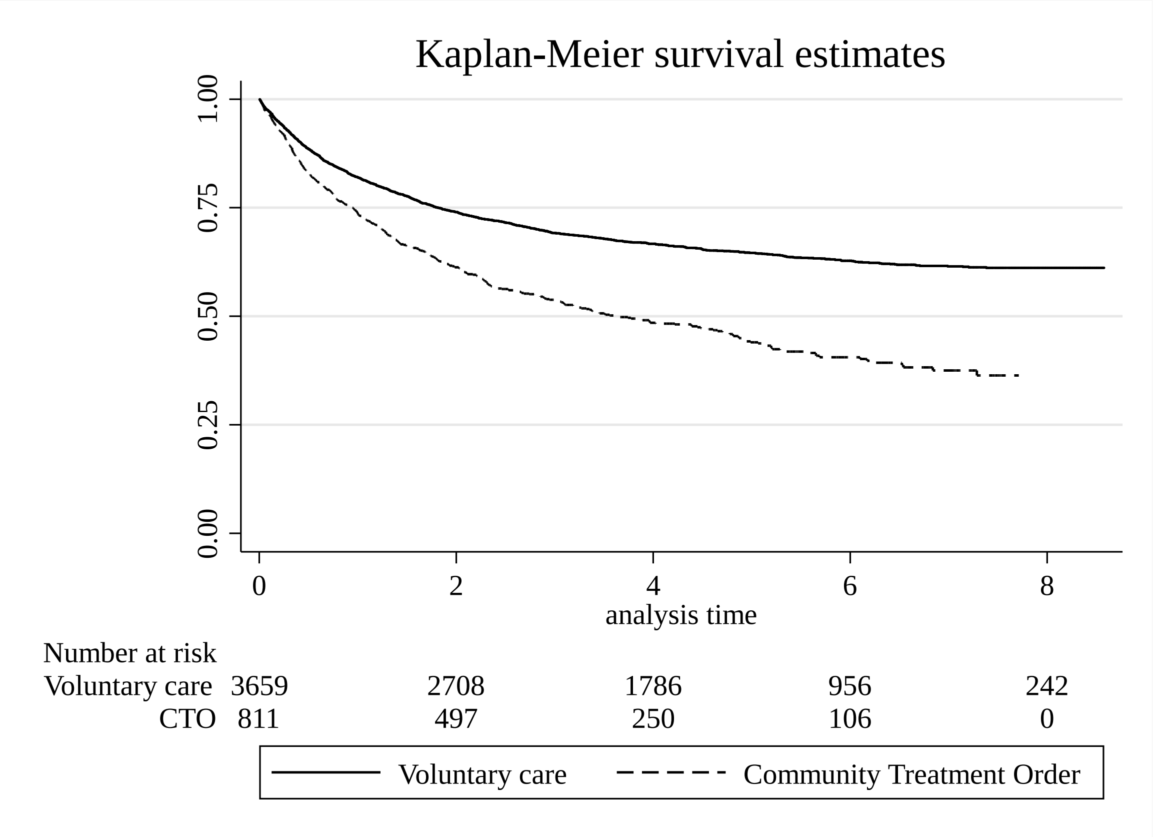

For peer review only - http://bmjopen.bmj.com/site/about/guidelines.xhtml

Community Treatment Orders: A controlled electronic case register study
Supplementary Material

Figure S2 Proportion of individuals with either a reduction or an increase in the amount of days spent in inpatient care in the 24 months before and after index admission, by CTO exposure (n = 4460).

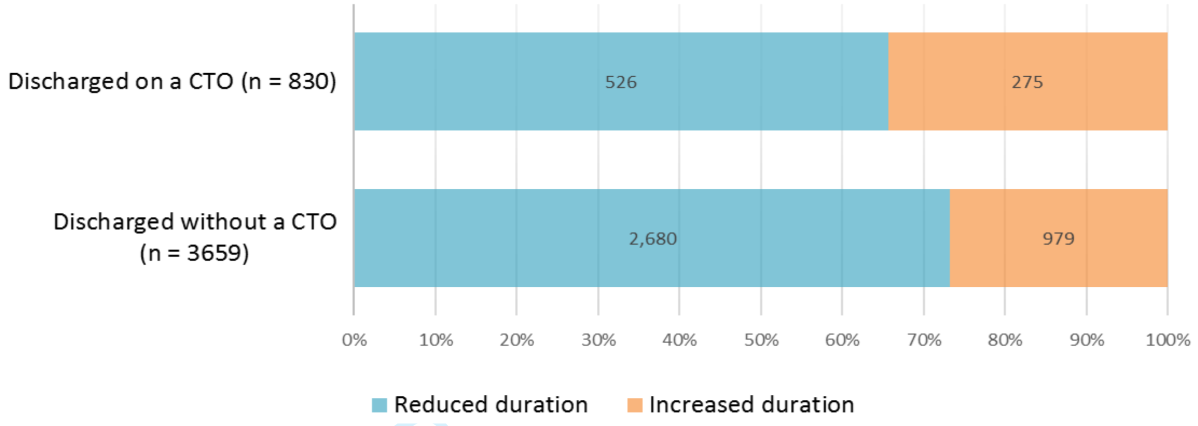

peer review only

STROBE Statement—checklist of items that should be included in reports of observational studies

	Item No	Recommendation
**Title and abstract**	1	(*a*) Indicate the study's design with a commonly used term in the title or the abstract p1,4
		(*b*) Provide in the abstract an informative and balanced summary of what was done and what was found p4
**Introduction**		
Background/rationale	2	Explain the scientific background and rationale for the investigation being reported p6-7
Objectives	3	State specific objectives, including any prespecified hypotheses p7 aims
**Methods**		
Study design	4	Present key elements of study design early in the paper p7 aims section
Setting	5	Describe the setting, locations, and relevant dates, including periods of recruitment, exposure, follow-up, and data collection p7-8 study population and data collection section
Participants	6	(*a*) *Cohort study*—Give the eligibility criteria, and the sources and methods of selection of participants. Describe methods of follow-up *Case-control study*—Give the eligibility criteria, and the sources and methods of case ascertainment and control selection. Give the rationale for the choice of cases and controls *Cross-sectional study*—Give the eligibility criteria, and the sources and methods of selection of participants p7-8 study population and data collection section
		(*b*) *Cohort study*—For matched studies, give matching criteria and number of exposed and unexposed *Case-control study*—For matched studies, give matching criteria and the number of controls per case *NA*
Variables	7	Clearly define all outcomes, exposures, predictors, potential confounders, and effect modifiers. Give diagnostic criteria, if applicable p8-10 Measures section
Data sources/ measurement	8*	For each variable of interest, give sources of data and details of methods of assessment (measurement). Describe comparability of assessment methods if there is more than one group p8-10 Measures section
Bias	9	Describe any efforts to address potential sources of bias p5 Strengths and limitations summary
Study size	10	Explain how the study size was arrived at p7-8 study population and data collection section
Quantitative variables	11	Explain how quantitative variables were handled in the analyses. If applicable, describe which groupings were chosen and why p9-11 Measures and Analyses sections

For peer review only - http://bmjopen.bmj.com/site/about/guidelines.xhtml

Statistical methods	12	(*a*) Describe all statistical methods, including those used to control for confounding<br>p9-11 Measures and Analyses sections
		(*b*) Describe any methods used to examine subgroups and interactions<br>NA
		(*c*) Explain how missing data were addressed<br>p10 missing data section
		(*d*) *Cohort study*—If applicable, explain how loss to follow-up was addressed<br>*Case-control study*—If applicable, explain how matching of cases and controls was addressed<br>*Cross-sectional study*—If applicable, describe analytical methods taking account of sampling strategy<br>p11 Analyses section
		(*e*) Describe any sensitivity analyses<br>p11 Analyses section
**Results**		
Participants	13*	(a) Report numbers of individuals at each stage of study—eg numbers potentially eligible, examined for eligibility, confirmed eligible, included in the study, completing follow-up, and analysed<br>p12 and Table 1
		(b) Give reasons for non-participation at each stage<br>NA (but see description of numbers and reasons for censored data p 12)
		(c) Consider use of a flow diagram<br>NA
Descriptive data	14*	(a) Give characteristics of study participants (eg demographic, clinical, social) and information on exposures and potential confounders<br>p 12
		(b) Indicate number of participants with missing data for each variable of interest<br>Table 1
		(c) *Cohort study*—Summarise follow-up time (eg, average and total amount)<br>p 12
Outcome data	15*	*Cohort study*—Report numbers of outcome events or summary measures over time
		Case-control study—Report numbers in each exposure category, or summary measures of exposure
		Cross-sectional study—Report numbers of outcome events or summary measures<br>*p 12, Table 1*
Main results	16	(*a*) Give unadjusted estimates and, if applicable, confounder-adjusted estimates and their precision (eg, 95% confidence interval). Make clear which confounders were adjusted for and why they were included<br>p12-14, Tables 1-3.
		(*b*) Report category boundaries when continuous variables were categorized<br>Table 1
		(*c*) If relevant, consider translating estimates of relative risk into absolute risk for a meaningful time period<br>NA
Other analyses	17	Report other analyses done—eg analyses of subgroups and interactions, and sensitivity analyses

For peer review only

		Online supplementary tables S2-3, figure S1
**Discussion**		
Key results	18	Summarise key results with reference to study objectives<br>p15 Main findings paragraph
Limitations	19	Discuss limitations of the study, taking into account sources of potential bias or imprecision. Discuss both direction and magnitude of any potential bias<br>p19 -20
Interpretation	20	Give a cautious overall interpretation of results considering objectives, limitations, multiplicity of analyses, results from similar studies, and other relevant evidence<br>p15-21
Generalisability	21	Discuss the generalisability (external validity) of the study results<br>p19-20
**Other information**		
Funding	22	Give the source of funding and the role of the funders for the present study and, if applicable, for the original study on which the present article is based<br>p2

*Give information separately for cases and controls in case-control studies and, if applicable, for exposed and unexposed groups in cohort and cross-sectional studies.

Note: An Explanation and Elaboration article discusses each checklist item and gives methodological background and published examples of transparent reporting. The STROBE checklist is best used in conjunction with this article (freely available on the Web sites of PLoS Medicine at http://www.plosmedicine.org/, Annals of Internal Medicine at http://www.annals.org/, and Epidemiology at http://www.epidem.com/). Information on the STROBE Initiative is available at www.strobe-statement.org.

For peer review only
